# Human SLFN5 and its *Xenopus Laevis* ortholog regulate entry into mitosis and oocyte meiotic resumption

**DOI:** 10.1038/s41420-022-01274-0

**Published:** 2022-12-08

**Authors:** Gianmatteo Vit, Alexander Hirth, Nicolas Neugebauer, Bianca N. Kraft, Gianluca Sigismondo, Anna Cazzola, Claudia Tessmer, Joana Duro, Jeroen Krijgsveld, Ilse Hofmann, Michael Berger, Harald Klüter, Christof Niehrs, Jakob Nilsson, Alwin Krämer

**Affiliations:** 1 Clinical Cooperation Unit Molecular Hematology/Oncology, German Cancer Research Center (DKFZ) and Department of Internal Medicine V, University of Heidelberg, Heidelberg, Germany; 2 Medical Faculty Mannheim, Institute for Transfusion Medicine and Immunology, Ruprecht-Karls University of Heidelberg, Mannheim, Germany; 3 The Novo Nordisk Foundation Center for Protein Research, Faculty of Health and Medical Sciences, University of Copenhagen, Copenhagen, Denmark; 4 Division of Molecular Embryology, DKFZ-ZMBH Alliance, Deutsches Krebsforschungszentrum (DKFZ), 69120 Heidelberg, Germany, and Institute of Molecular Biology (IMB), Mainz, Germany; 5 Division of Proteomics of Stem Cells and Cancer, German Cancer Research Center (DKFZ), Heidelberg, Germany; 6 Genomics and Proteomics Core Facility, Unit Antibodies, German Cancer Research Center (DKFZ), Heidelberg, Germany; 7 The Lautenberg Center for General and Tumor Immunology, The Hebrew University of Jerusalem, Hadassah Medical School, Jerusalem, Israel

**Keywords:** Checkpoints, Kinases

## Abstract

The *Schlafen* gene family was first described in mice as a regulator of thymocyte development. Further studies showed involvement of human orthologs in different processes related with viral replication, cellular proliferation, and differentiation. In recent years, a new role for human *Slfn11* in DNA replication and chromatin remodeling was described. As commonly observed in many gene families, *Slfn* paralogs show a tissue-specific expression. This made it difficult to reach conclusions which can be valid in different biological models regarding the function of the different Schlafen proteins. In the present study, we investigate the involvement of SLFN5 in cell-cycle regulation and cell proliferation. A careful analysis of SLFN5 expression revealed that SLFN5 is highly expressed in proliferating tissues and that the protein is ubiquitously present in all the tissues and cell line models we analyzed. Very interestingly, SLFN5 expression oscillates during cell cycle, peaking during S phase. The fact that SLFN5 interacts with protein phosphatase 2A and that SLFN5 depletion causes cell cycle arrest and cellular apoptosis, suggests a direct involvement of this human paralog in cell cycle progression and cellular proliferation. We substantiated our in vitro and *in cellulo* results using *Xenopus laevis* oocytes to show that mRNA depletion of the unique *Slfn* gene present in *Xenopus*, whose protein sequence shares 80% of homology with SLFN5, recapitulates the phenotype observed in human cells preventing the resumption of meiosis during oocyte development.

## Introduction

*Schlafen (Slfn)* genes belong to a conserved metazoan gene family involved in cell proliferation and differentiation [1]. Although previous work has shown that murine *slfn* genes are involved in cell cycle regulation, very little is known about the contribution of human *SLFNs* to cell cycle control. Initially, murine *slfn1* was described to control cell cycle entry in thymocytes [2]. Also, a role for murine *slfn2* in the maintenance of T cell quiescence has been suggested [3]. Furthermore, it has been shown that human *SLFN11* is involved in the regulation of S phase progression, binds to DNA replication forks under stress conditions and blocks their progression by chromatin remodeling [4, 5]. However, most cell lines used in biomedical research are devoid of SLFN11 [4]. Thus, it remains unknown whether other *SLFNs* substitute for *SLFN11* or regulate other processes involved in cell cycle control in human cells. Many proteins involved in cell cycle regulation show evolutionary conserved structures, periodic cell cycle-dependent expression, and are universally expressed in every cell type. The cell cycle is orchestrated by reversible phosphorylation events which are orchestrated by protein kinases and protein phosphatases. Protein phosphatase 2A (PP2A), one of the most abundant serine/threonine protein phosphatases (PPPs), plays a pivotal role in interphase maintenance by counteracting the activity of cyclin B/CDK1 and further opposes CDK1 activity at mitotic exit [6, 7].

Here, we show that SLFN5, which shares a high degree of sequence homology with SLFN11, is ubiquitously expressed in proliferating cells and plays a role in the regulation of the transition from S to G_2_ phase of the cell cycle. Consistently, we find that the protein levels of SLFN5 are regulated during the cell cycle peaking in S phase. In human cells SLFN5 associates with PP2A-B55 in a phosphorylation dependent manner. We demonstrate that the phosphorylation status of SLFN5 influences PP2A-B55α binding and normal cellular proliferation. Furthermore, we show that the Xenopus laevis ortholog of human SLFN5 is involved in meiotic resumption of G_2_-arrested Xenopus oocytes, suggesting that the cell cycle function of SLFN5 is conserved throughout evolution.

## Results

**Fig. 1:**
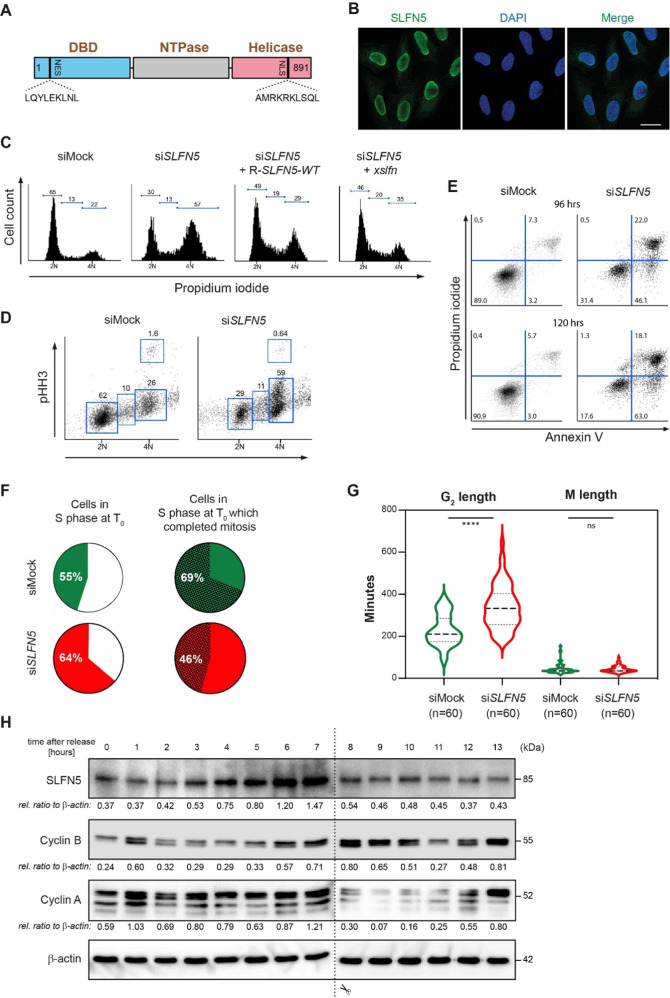
SLFN5 loss-of-function results in G_2_/M arrest. **A** Structure of SLFN5. SLFN5 consists of three hitherto uncharacterized domains: a N-terminal DNA binding domain (DBD), a C-terminal DNA/RNA helicase domain and a nucleoside triphosphate hydrolase domain (NTPase). A canonical nuclear localization signal (NLS) and a nuclear export signal (NES) are present at the C- and N-terminus, respectively. **B** Immunostaining of U2OS cells with #111/1 SLFN5 monoclonal antibody reveals nuclear localization of SLFN5. DNA is stained with Hoechst. Scale bar represents 20 µm. **C**, **D** Downregulation of SLFN5 in U2OS cells by RNAi for 48 h leads to G_2_/M arrest. G_2_/M arrest after siRNA-mediated SLFN5 knockdown can be partially rescued by a siRNA-resistant (R-)*SLFN5* construct and its *Xenopus laevis* ortholog *xslfn*. Cells were harvested 48 h after transfection with siMock or si*SLFN5*. Rescue experiments were performed by concomitant transfection of si*SLFN5* and R-*SLFN5* or *xslfn*. Cell cycle analysis was performed by combined propidium iodide (PI)/phosho-histone H3 (pHH3) FACS staining. The pictures show a representative experiment from three independent experiments. **E** Downregulation of SLFN5 in U2OS cells by RNAi leads to apoptosis. Cells were harvested 96 and 120 h after transfection with siMock or si*SLFN5*. The fraction of apoptotic cells was determined after Annexin V staining by FACS analysis. The pictures show a representative experiment from three independent experiments. **F**, **G** Cell cycle progression of SLFN5 depleted cells assessed by time-lapse microscopy over 12 h. Pie charts indicate percentages of cells in S phase at T_0_ (start of filming) (**F**, left) and cells in S phase at T_0_ which successfully divided (**F**, right), respectively. Violin plots statistically illustrate length of both G_2_ and M phase of SLFN5-depleted cells **G**. Green and red colours identify siMock-treated and si*SLFN5*-treated cells, respectively. The graphs statistically illustrate the results from three independent experiments. **H** Western blotting of SLFN5 expression during cell cycle in U2OS cells. U2OS cells were arrested at G_1_/S phase by double-thymidine treatment and released from arrest by thymidine washout. Samples were collected for Western blotting in 60 min intervals, until cells reached the mitotic state (12–13 h). Cyclin B and Cyclin A were used as markers for G_2_/M and interphase, respectively. β-actin was used as loading control. The Western blot shows a representative experiment from three independent experiments.

Whereas murine SLFNs have been described to control cell cycle progression in T cells [2, 3, 8], only little is known on a potential role for human SLFN proteins in cell cycle regulation. RNA expression data from the Human Protein Atlas suggest ubiquitous expression of *SLFN5*, *11* and *12,* whereas the expression of other human *SLFN* genes seems to be more tissue-specific (https://www.proteinatlas.org/humanproteome/cell). After raising monoclonal antibodies against SLFN5 and SLFN12 (Figures S1A–D) and using a commercial antibody against SLFN11, we assessed by Western blotting their protein expression levels in several cell lines of diverse tissue types and healthy primary human tissues. Only SLFN5 was ubiquitously expressed in proliferating tissues at the protein level (Figures S2A, B). Computational analysis revealed that SLFN5 is composed of a N-terminal DNA binding domain (aa 191–308), a P-loop NTPase domain (aa 574–657) and a C-terminal helicase domain (aa 839–886) (Fig. 1A and https://www.ncbi.nlm.nih.gov/Structure/cdd/wrpsb.cgi?seqinput=NP_659412.3). Immunostaining and cellular fractionation experiments in U2OS cells revealed that human SLFN5 localizes to the nucleus (Fig. 1B and S3A), like its murine orthologue [9].

Recently, human SLFN11 was shown to regulate S phase progression and chromatin remodeling [4, 5]. To explore the cellular function of human SLFN5, we depleted endogenous SLFN5 in U2OS cells using a pool of four different siRNAs (si*SLFN5*) (Figure S3B) and analyzed cell cycle progression by flow cytometry. After si*SLFN5* transfection, 57% of the cells were arrested in G_2_/M phase as compared to only 22% of mock-transfected cells. This arrest could be partially rescued by co-transfection of a WT siRNA-resistant (R-) *SLFN5* construct. Interestingly, the *Xenopus laevis* ortholog of *SLFN5* (*xslfn*), whose protein product shares about 85% of its primary amino acid sequence with human SLFN5, was able to almost completely rescue the G_2_/M arrest (Fig. 1C, D and S3B). SLFN5-depleted cells became apoptotic after 96 h, as shown by an increased fraction of Annexin V positive cells (Fig. 1E). To substantiate these results and monitor cell cycle progression of SLFN5-depleted cells, siSLFN5-treated U2OS cells constitutively expressing GFP-PCNA were monitored for 12 h by live-cell microscopy (Figure S3C and supplementary videos). At the beginning of the experiment, 64% of si*SLFN5*-treated cells were in S phase as determined by the presence of PCNA foci, compared to 55% of siMock-treated cells in S phase. During the 12 h of recording only 46% of the original population of si*SLFN5*-treated cells in S phase (64%) entered mitosis and divided. In contrast 69% of the siMock-treated cells in S phase (55%) at the beginning of the filming successfully completed cell division (Fig. 1F). We analyzed the length of both G_2_ (time from disappearance of PCNA replicative foci to nuclear envelope breakdown (NEBD)) and M phase (time from NEBD to anaphase) and observed a statistically significant delay in G_2_-phase progression of SLFN5-depleted cells while no significant changes in M phase length could be detected (Fig. 1G). Together, these findings suggest that SLFN5 is required for the normal progression through S and G_2_ phases. To investigate if SLFN5 protein levels oscillates during cell cycle progression, we released U2OS cells from a double thymidine-induced G_1_/S arrest and collected samples at one-hour intervals. We found that SLFN5 protein levels fluctuate during cell cycle, peaking at 6–7 h after release, which corresponds to late S or G_2_ (Fig. 1H).

**Fig. 2:**
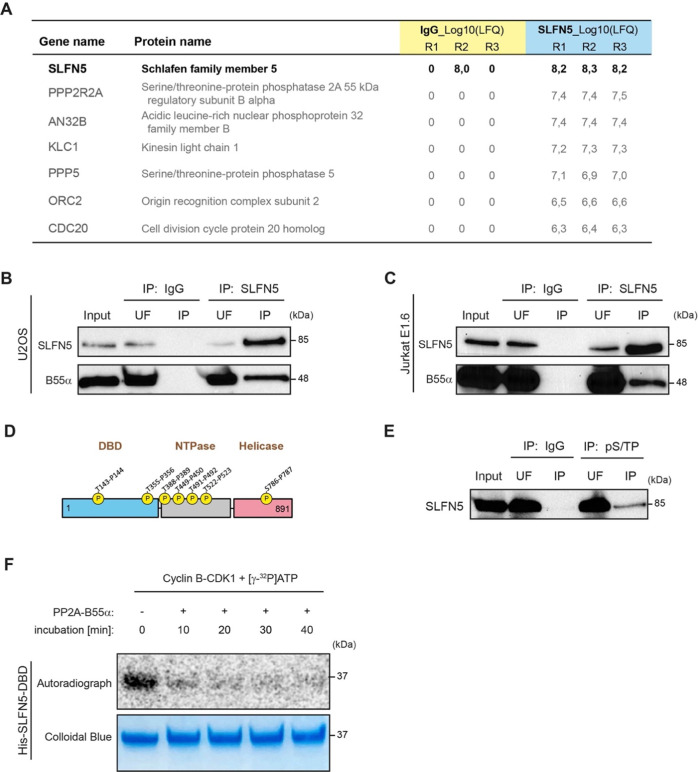
PP2A-B55α interacts with SLFN5 and dephosphorylates its CDK minimal consensus sequences. **A** Table summarizing the strongest interactors identified by mass spectrometry after SLNF5 immunoprecipitation. For each protein, the log10 values of the label-free quantification intensities (LFQ) in the three replicates of the IgG-IP and SLNF5-IP are reported. **B**, **C** Validation of SLFN5 - B55α interaction in U2OS and Jurkat E1.6 cells by co-immunoprecipitation followed by Western blotting. #112/5/6 anti-SLFN5 was used to co-immunoprecipitate endogenous SLFN5 and its interactors. Immunoprecipitation with an irrelevant mouse IgG antibody served as a negative control (control IP). B55α recovery was confirmed using an antibody against the regulatory subunit B55α. UF, unbound fraction. The Western blot shows a representative experiment from three independent validations. **D** SLFN5 contains a cluster of five TP motifs in the DBD and NTPase domain, and single TP (T143-P144) and SP (S786-P787) motifs at the N-terminus and C-terminus, respectively. **E** SLFN5 is phosphorylated at T/SP minimal CDK consensus sequences. An anti-pT/SP antibody was used for immunoprecipitation in U2OS cells. The membrane was probed with #112/5/6 anti-SLFN5. Immunoprecipitation with an irrelevant mouse IgG antibody served as a negative control. The Western blot shows a representative experiment from three independent experiments. **F** SLFN5-DBD TP sites are phosphorylated by Cyclin B-CDK1 and dephosphorylated by PP2A-B55α. Timecourse of SLFN5-DBD dephosphorylation. Comassie staining serves as loading control. The Radiograph shows a representative experiment from three independent experiments.

To understand at a molecular level how SLFN5 regulates cell cycle progression at the G_2_/M transition, we next sought to identify SLFN5 interaction partners. We immunoprecipitated endogenous SLFN5 and subsequently analyzed co-immunoprecipitated proteins by mass spectrometry (Co-IP/MS). Several proteins involved in cell cycle regulation, including serine/threonine-protein phosphatase 2 A regulatory subunit B55α (PP2A-B55α), serine/threonine-protein phosphatase 5 (PP5), origin recognition complex subunit 2 (ORC2) and the APC/C activator protein CDC20, were found to interact with SLFN5 (Fig. 2A; Supplementary Excel Files 1 and 2). PP2A-B55 plays a pivotal role in the regulation of mitotic entry by counteracting the interphase activity of cyclin dependent kinases (CDK) [6, 7, 10–12]. Therefore, we decided to further focus on the validation and functional analysis of the interaction between SLFN5 and PP2A regulatory subunit B55α. Co-IP followed by Western blotting in both suspension (Jurkat E6.1) and adherent (U2OS) cells validated the interaction between SLFN5 and B55α (Fig. 2B, C).

**Fig. 3:**
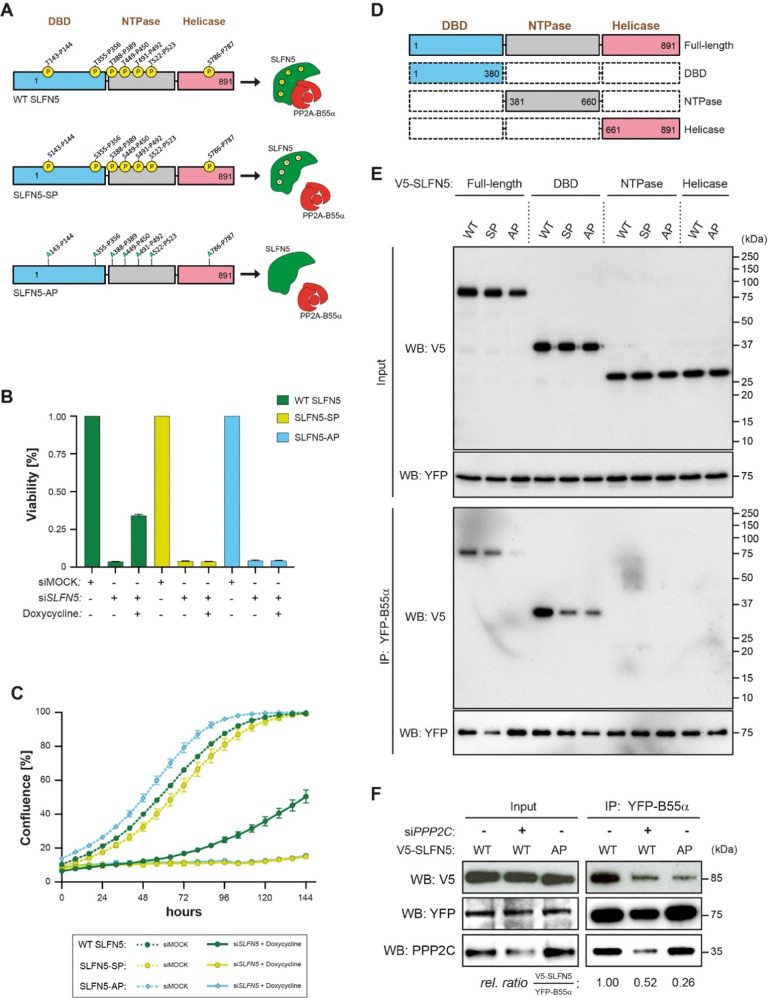
SLFN5 TP sites are essential for U2OS cell proliferation and viability and required for B55α binding. **A** Model of SLFN5 regulation and binding to PP2A-B55α. SLFN5 minimal TP consensus sequences can be dephosphorylated by PP2A-B55α (upper panel). Serine-to-Threonine substitution reduces PP2A-B55α binding to SLFN5 (middle panel). A non-phosphorylatable SLFN5 version where threonine residues have been replaced by alanine prevents PP2A-B55α binding and SLFN5 activity (lower panel). **B**, **C** Cell viability and cell growth are impaired in SLFN5-depleted U2OS cells and can be rescued by expression of RNAi resistant WT SLFN5. Cells were treated for three days with RNAi against *SLFN5* or Luciferase and grown for further 9 days in presence or absence of Doxycycline. Cell viability was assessed by SRB assay (**B)**, or cell confluence was monitored over 6 days under the same conditions by Incucyte growth assay to assess cell proliferation (**C)**. The graphs statistically illustrate the results from three independent experiments (**B**, **C)**. **D**, **E** B55α interacts with the SLFN5 DNA binding domain (DBD). WT, SP, and AP variants of V5-tagged full length SLFN5 and its single domains were transiently transfected into inducible YFP-B55α-HeLa cells. YFP-B55α expression was induced by addition of doxycycline. Immunoprecipitation was performed using an antibody to GFP. SLFN5 recovery was probed by Western blotting using a V5-antibody. The Western blot shows a representative experiment from three independent experiments (**E)**. **F** Depletion of PP2A catalytic subunits α and β (siPPP2C) in inducible YFP-B55α-HeLa cells impairs WT SLFN5 recovery. Cells were treated with RNAi oligomers targeting both isoforms of PPP2C for 48 h and WT SLFN5 recovery was assessed by Western blotting using V5-antibody after pulldown of the YFP-tagged B55α regulatory subunit of PP2A. YFP-B55α expression was induced by treatment with Doxycycline. The Western blot shows a representative experiment from three independent experiments.

SLFN5 contains a cluster of five Thr-Pro (TP) minimal CDK consensus sequences in its DBD-NTPase domains, one TP site in the DBD domain and a single Ser-Pro (SP) sequence in the C-terminal helicase domain (see Fig. 2D for schematics). To determine if SLFN5 is phosphorylated on S/TP sites in cells, we immunoprecipitated the U2OS nuclear phospho-S/TP (pT/SP) proteome using a monoclonal antibody specific for pT/SP sequences and identified SLFN5 among the immunoprecipitated proteins (Fig. 2E). To establish if CDKs could be responsible for SLFN5 phosphorylation, we assessed SLFN5-DBD (which will be demonstrated later in this work as being the domain interacting with PP2A- B55α - see Fig. 3C) phosphorylation by CDK1-CyclinB1. Recombinant SLFN5 DBD was incubated with radioactive ATP and CDK1-Cyclin B1 which revealed phosphorylation of the DBD (Fig. 2F, first lane). We next investigated if the SLFN5 DBD phosphorylated by CDK1-Cyclin B1 is a substrate of PP2A-B55α. We incubated phosphorylated DBD with purified PP2A-B55α enzyme and monitored over time the removal of radioactive ^32^P. Already after 10 min PP2A-B55α was able to effectively dephosphorylate SLFN5-DBD as assessed by autoradiography (Fig. 2F) suggesting that SLFN5 is both an interaction partner and substrate of PP2A-B55α.

It has been previously reported in both yeast and humans that PP2A-B55α has a preference for dephosphorylating TP over SP phosphorylation sites [13, 14]. This preference, together with its CDK-counteracting function are important for coordinated phosphorylation/dephosphorylation events during cell cycle progression [13, 15]. We next asked if the phosphorylation and efficient dephosphorylation of these sites is important for SLFN5 function and binding to PP2A-B55α. We therefore constructed mutant forms of SLFN5 where all TP sites were mutated to either SP sites (SLFN5-SP) or AP sites (SLFN5-AP) (see Fig. 3A for schematics). We generated stable U2OS cell lines expressing IRES-GFP-inducible, siSLFN5 resistant WT SLFN5, SLFN5-SP and SLFN5-AP, respectively. Cell growth could be partially rescued by expression of an RNAi resistant WT SLFN5 form after endogenous SLFN5 depletion, while both SP and AP variants could not compensate for endogenous SLFN5 downregulation (Fig. 3B, C and S3D). This suggests that phosphorylation as well as efficient dephosphorylation of SLFN5 is required for cell proliferation.

PP2A-B55α is inhibited by the ENSA and Arpp19 proteins that binds in a phosphorylation dependent manner to the phosphatase [11]. This prompted us to investigate if the SLFN5 phosphorylation sites contribute to PP2A-B55α binding. To determine this, inducible stable YFP-B55α HeLa cells were transiently transfected with V5-SLFN5 constructs (V5-SLFN5 WT, V5-SLFN5-SP and V5-SLFN5-AP) (Fig. 3A). After immunoprecipitation of YFP-B55α, full-length V5-SLFN5 and single domains thereof (V5-SLFN5^DBD^, V5-SLFN5^NTPase^ and V5-SLFN5^helicase^) (Fig. 3D), each as WT, SP or AP variants, were assessed for recovery by V5 Western blotting. As shown in Fig. 3E, the N-terminal DNA binding domain of SLFN5 mediates the binding to B55α and mutation of wildtype TP to SP or AP sites consistently impaired B55α binding. This indicates that the SLFN5 phosphorylated TP sites directly contribute to PP2A-B55 binding. Given this, we speculated whether the catalytic subunit of PP2A-B55 contributes to SLFN5 binding. To test this, the PP2A catalytic subunits PPP2Cα and PPP2Cβ were knocked down by siRNA transfection (si*PPP2C*) in inducible YFP-B55α HeLa cells. Subsequently, YFP-B55α immunoprecipitates were assessed for SLFN5 co-immunoprecipitation by Western blotting. Similar to V5-SLFN5-AP, PPP2C depletion led to a marked reduction in SLFN5 recovery (Fig. 3F).

To summarize, SLFN5 appears to be a target of CDK kinases and a substrate of PP2A-B55 and that the phosphorylation and dephosphorylation of SLFN5 is involved in the regulation of cell viability.

**Fig. 4:**
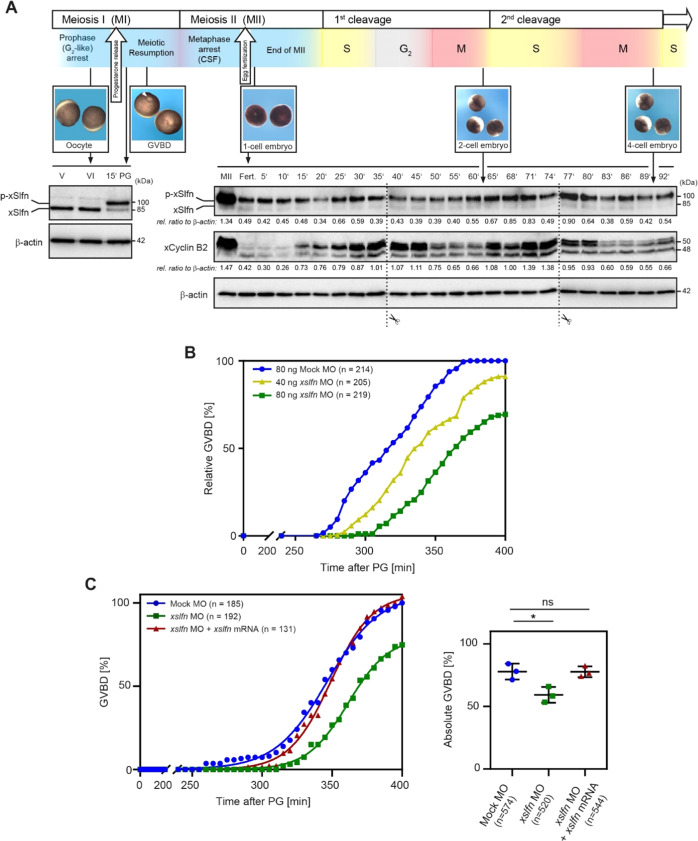
xSlfn expression oscillates during *X. laevis* embryonic development and xSlfn loss-of-function delays oocyte meiotic resumption. **A** Schematic representation of *X. laevis* oocyte development from stage V to metaphase arrest of meiosis II (MII). Mature stage VI oocytes are arrested in a “G_2_-like”-state in prometaphase of the first meiotic division (MI). Only after progesterone (PG) treatment they complete MI and begin MII until a second arrest at metaphase of MII (cytostatic factor arrest, CSF arrest) blocks further progression of development for a second time. Developmental progression is resumed at fertilization, when completion of the second meiotic division and start of first embryonic cell divisions take place (upper panel). For immunoblotting, oocytes and embryos were collected at different developmental stages and expression of xSlfn and phosphorylated xSlfn (p-xSlfn) was assessed using an antibody to xSlfn. Fertilization was assessed by the appearance of cortical rotation. Cyclin B2 was used as a marker for G_2_ and M phase. β-actin was used as loading control. The Western blot shows a representative time course from three independent collections. **B**, **C** xSlfn loss-of-function delays meiotic resumption. Microinjection of antisense Morpholino targeting the 5′-UTR of *xslfn* delays the progression from G_2_-phase/prophase of MI to metaphase of MII after progesterone treatment in a dose-dependent manner (**B**) and can be rescued by co-injection of non-targetable *xslfn* mRNA (**C**). Oocytes injected with Mock-Morpholino (MO) served as a control. In **C**, the left panel shows a representative experiment from three independent experiments. The right panel depicts the overall number of oocytes reaching GVBD. Each data point represents one independent replicate. *, *P* < 0.05; ns, not significant (Two-sided Student´s *t*-test).

*Schlafen* genes emerged early during metazoan evolution and underwent several duplication events during speciation. Higher vertebrates have many *Slfn* paralogs. Humans and mice carry six and ten *Slfn* genes, respectively, whereas the majority of fish species and *Xenopus laevis* have only one *Slfn* gene, *slfn13* according to *Xenbase*, referred to here as *xslfn* [16]. As *X. laevis* oocytes represent an established model to investigate the G_2_/M transition of the cell cycle and given the high level of conservation between xSlfn and SLFN5 protein sequences, we next determined xSlfn protein expression during *Xenopus* development after raising a mouse monoclonal antibody to xSlfn (Figures S4A, B). Antibody specificity was tested using Morpholino (MO) oligomers targeting the 5 ´-UTR of *xslfn* mRNA to downregulate xSlfn protein expression in *X. laevis* embryos. In immunoblots, in addition to the xSlfn band at 85 kDa, a band at ~100 kDa consistently decreased after MO-mediated xSlfn depletion (Figure S4C), suggesting that xSlfn was post-translationally modified. Treatment of whole-embryo protein extracts with λ-phosphatase confirmed the presence of a phosphorylated form of xSlfn (p-xSlfn) (Figure S4D). To systematically analyze xSlfn expression during *X. laevis* embryonic development, unfertilized oocytes, fertilized eggs and developing embryos at 1-cell and 2-cell stages were collected and protein expression was assessed by Western blotting. Non-phosphorylated xSlfn was expressed from the earliest stages of oocyte development until G_2_/prophase arrest of the first meiotic division (MI) at oocyte stage VI (Fig. 4A, left panel). Interestingly, already 15 min upon progesterone treatment, xSlfn became phosphorylated. xSlfn phosphorylation constantly persisted until metaphase arrest of the second meiotic division (MII) (Fig. 4A, right panel, and data not shown), where xSlfn expression reached its maximum. Upon fertilization, p-xSlfn expression oscillated, with a first minimum at 20 min post fertilization (mpf) and a subsequent maximum at 30 mpf, concomitant with increasing cyclin B2 levels at first cell division. p-xSlfn expression decreased again at 40 mpf, shortly before cyclin B2 reached its highest levels at 45 mpf. Cyclin B2 was subsequently degraded at mitotic exit between 50 and 60 mpf. At this point the first embryonic division had been completed. During the shorter second embryonic cell cycle, p-xSlfn and cyclin B2 expression and degradation proceeded in parallel, decreasing again at 80 mpf, before cell division (Fig. 4A, right panel).

Meiotic resumption, i.e. the release of oocytes from G_2_ arrest, is initiated by progesterone-mediated inhibition of protein kinase A (PKA) activity, which leads to translation of maternal mRNAs, resulting in cyclin B-cdc2 (MPF, maturation promoting factor) activation, progression through first and second meiosis (MI and MII) and arrest at metaphase of MII by cytostatic factor (CSF) [17], (schematic representation in Fig. 4A). To test whether xSlfn is also involved in G_2_/M transition, immature *X. laevis* stage VI oocytes were microinjected with either 40 or 80 ng *xslfn* Morpholino (*xslfn* MO) or with 80 ng of a control Mock Morpholino (Mock MO). Western blotting confirmed successful xSlfn knockdown in oocytes (Figure S4E). 24 h following injection, oocytes were treated with 10 µg/ml progesterone to induce release from G_2_ arrest and to initiate the onset of germinal vesicle breakdown (GVBD) as a marker of MII metaphase arrest (CSF-arrest). Progesterone-treated oocytes injected with *xslfn* MO reached GVBD consistently later than Mock MO-injected oocytes in a dose-dependent manner (Fig. 4B). Moreover, 77.8 ± 5.2% of the Mock MO-injected oocytes, but only 59.3 ± 5.1% of the *xslfn* MO-injected oocytes reached GVBD within the observation period. Both, delay of GVBD onset and the total number of oocytes reaching GVBD (77.7% ± 3.5%) were efficiently rescued by co-injection of non-targetable FLAG-tagged *xslfn* mRNA (Fig. 4C).

## Discussion

The *Schlafen* gene family is highly redundant in higher vertebrates and its function has been ascribed to different processes so far. In this study we have shown that SLFN5 is a ubiquitously expressed human SLFN paralog in actively replicating cells. In contrast, SLFN11 and SLFN12 show a higher degree of tissue specificity. Protein expression levels of SLFN5 and its *X. laevis* ortholog oscillate during somatic cell cycle in U2OS cells and first cleavages of *Xenopus* embryos, respectively, with highest expression levels at the S and G_2_/M transition in U2OS cells and in G_2_-like (MI-prophase) and CSF-arrested *X. laevis* oocytes (MII-metaphase). We show that the phosphorylation and likely rapid dephosphorylation of SLFN5 plays a role in regulating cell proliferation and likely in the progression through S and G_2_. SLFN11, which shares the highest degree of homology with SLFN5 among human SLFNs, also regulates cell cycle transition at S phase. It binds to chromatin upon replication stress and induces replication block and cell death in an ATR-CHK1 independent way [4, 5]. Future research should determine if SLFN5 functions is also involved in DNA repair and if the observed G_2_ delay could be explained as a consequence of DNA damage checkpoint activation.

Since our data shows that PP2A-B55α binds more efficiently to the phosphorylated form of SLFN5, this argues for a possible role of CDK in regulating a rapid transition between the phosphorylated and unphosphorylated form of SLFN5.

How PP2A-B55α recognizes and binds to its substrates is still unclear. Hertz and colleagues demonstrated that a conserved short linear motif is required for binding to the PP2A-B56 holoenzyme [18]. For the B55 regulatory subunit it was shown that basic residues flanking a minimal CDK consensus sequence promoted B55 dephosphorylation [15, 19]. However, a 20 amino acid polybasic region containing three arginine residues was identified as crucial for PP2A-B55 binding to mCRTC3 and recently a short linear motif (HxRVxxV) could be identified as mediator of B55α binding in p107, a member of the Retinoblastoma tumor suppressor protein family [20, 21]. SLFN5 contains only a single small basic motif (RKRK) at its C-terminus and some additional pairs of basic amino acids which are distributed along the protein. Thus, SLFN5-B55α interaction is unlikely to be mediated by similar basic patch interactions. More likely, yet unidentified elements play a role in the recognition of SLFN5 by PP2A-B55α. B55α immunoprecipitation reproducibly recovered SLFN5 through binding to its DBD. Furthermore, our data suggest that phosphorylated TP sites stimulates binding possibly via direct interaction with the catalytic subunit. This leaves a broad spectrum of possibilities open on how B55α recognizes its substrates, and we can surmise that not only the primary sequence of the target protein, but post-translational phosphorylation may influence the interaction.

We finally gained first insight into the conserved function of SLFN5 across vertebrates by investigating the function of its *X. laevis* ortholog. The finding that Morpholino-mediated downregulation of xSlfn delayed the time needed to reach GVBD further supports the involvement of SLFN5 in the regulation of the G_2_/M transition and the evolutionary conserved function of this protein.

There are several limitations of the present work which will require further investigation. First, the mechanistic explanation on how SLFN5 participates in the regulation of cell cycle progression and the molecular mechanism underlying the observed phenotype after SLFN5 and xSLFN depletion have still to be investigated. To this end, it will be of particular interest to understand if SLFN5 mechanism of action in part recapitulates that of SLFN11. Second, the interaction between SLFN5 and PP2A-B55α and the phosphorylation state of SLFN5 remain to be understood in further detail and how this relates to the cell cycle defects we observed. This requires the identification of SLFN5 mutants that are specifically defective in PP2A-B55α binding. Furthermore, an in-depth investigation in in vivo assays will be crucial to establish both enzyme specificity and concentration required for SLFN5 phosphorylation and dephosphorylation.

## Method details

### Human cell culture, cell synchronization and transfection

Human cell cultures were maintained at 37 °C in a humidified 5% CO_2_ environment. Cells were cultured in the appropriate medium supplemented with the necessary nutrients as indicated by the American Type Culture Collection (ATCC), Manassas, Virginia, USA, or by the German Collection of Microorganisms and Cell Cultures GmbH (DMSZ), Leibniz Institute, Braunschweig, Germany. Cell media were supplemented with 10% Fetal Bovine Serum (FBS) and Penicillin-Streptomycin (P/S), 10,000 U/ml. Media used for RNAi experiments were devoid of Penicillin-Streptomycin. Multiplex cell contamination (McCT) and Multiplex human cell line authentication tests (MCA) were performed by Multiplexion GmbH (Friedrichshafen, Germany).

U2OS cells were synchronized in G_1_/S phase by double thymidine (Sigma-Aldrich) block (2.5 mM): cells were grown for 24 h in thymidine-enriched medium. After 24 h thymidine wash-out was performed and fresh medium was added to the cells for 9 h. Successively, thymidine was added to the cells for 15 h prior to thymidine washout and release in normal medium.

RNAi oligomers against SLFN5, BB5α regulatory subunit of PP2A (PPP2R2A), catalytic subunits α and β of PP2A (PPP2CA, PPP2CB) and non-targeting control siRNA were purchased from Dharmacon^TM^. RNAi oligomers against the catalytic subunits α and β of PP2A (PPP2CA, PPP2CB) were purchased from Ambion, Life Technologies. Oligomers were transfected at a 20 nM final concentration each using Lipofectamine^TM^ RNAiMAX Transfection Reagent (Thermo Fisher Scientific^TM^), following manufacturer ´s instructions. All RNAi oligomer sequences are listed in Table S1.

Transient transfection of plasmid DNA was performed using the jetPRIME^®^ transfection reagent (Polyplus^TM^), according to manufacturer ´s recommendations. 0.5 to 1 μg of plasmid DNA was used for transfection and cells were incubated for 6 to 48 h.

Cotransfection of siRNA oligomers and plasmid DNA for rescue experiments was performed using Lipofectamine^TM^ 2000 Transfection Reagent (Thermo Fisher Scientific^TM^), according to manufacturer ´s protocol. YFP-B55α gene expression in HeLa cells was induced by addition of 5 ng/ml Doxycycline for 12 h.

### Mouse cell culture

Hybridoma cell cultures were maintained in HAT Medium (Gibco) supplemented with 10% FBS, 2 mM Glutamine, 1% Penicillin/Streptomycin, MEM amino acids solution (Thermo Fisher Scientific^TM^) and 0.5 mM sodium pyruvate. Cells were subsequently weaned off serum in RPMI supplemented with 10% FBS and later 5% FBS.

### Amphibian cell culture

Amphibian cells were maintained at 26 °C in a humidified 6% CO_2_ environment. Cells were cultured in Amphibian serum-free medium supplemented with 10% FBS, as follows: 200 ml Mammalian Serum Free Basic Medium (MSF) (IMDM 1 pkg, P/S 10 ml, NEAA 10 ml, Insulin 10 ml, 2-Me 1 ml, Primatone 3 ml, NaHCO3 3.02 g (for 1 L medium)) supplemented with 5 ml P/S and 500 μl kanamycin. Media and phosphate buffered saline (PBS) solution were adapted to the amphibian osmolarity of 190 mOs/kg. pH was adapted to 7.0 with 10 N NaOH. Medium was filtered through 0.2 μm filter and stored at 4 °C. Multiplex cell contamination tests (McCT) were performed by Multiplexion GmbH (Friedrichshafen, Germany; www.multiplexion.com).

### *Xenopus laevis* maintenance and husbandry

All *Xenopus* experiments were approved by the state review board of Baden-Württemberg (Germany) and performed according to federal and institutional guidelines. *X. laevis* females were obtained from EXRC (Portsmouth, UK) and Nasco (Fort Atkinson, USA) and kept in a *Xenopus* facility designed by Tecniplast. *X. laevis* are kept at 18 °C with a light/dark cycle of 12 h/12 h. *X. laevis* females were killed in a bath of MS-222 (Sigma-Aldrich).

### Development of monoclonal antibodies against SLFN5, SLFN12, and xSlfn

Generation of monoclonal antibodies was performed according to the principles of Köhler and Milstein’s hybridoma technology [22]. Mouse immunization was performed according to the following protocol: on day 1, 4 and 7, 20 μg of recombinant antigen or in vitro synthetized peptide antigen (PSL GmbH, Heidelberg), N-terminal-conjugated through a cysteine residue to Keyhole Limpet Hemocyanin (KLH) were used to immunize BALB/c and C57BL/6 N mice. For SLFN5 (Uniprot Q08AF3) the protein fragment aa660–891, for SLFN12 (Uniprot Q8IYM2) the peptide VVDAGKVTLGTQQRQE and for xSlfn (Uniprot Q6NTK7) the protein fragment aa1–480 was used. To enhance the immune answer, 100 μl of Freund’s Complete Adjuvant (Santa Cruz Biotechnology) were injected in the hind leg of each mouse. Boost injections were done with Freund’s Incomplete Adjuvant followed by injection with buffer only. On day 8 anti-sera were tested for specific immune reaction on immunoblotting membrane blotted with protein lysate obtained after overexpression of the protein of interest in HEK-293T cells.

The fusion procedure to originate hybridoma cell clones was performed as follows: popliteal lymph nodes were surgically removed from the knee throat of the mouse under sterile conditions and placed in RPMI medium (Gibco). Lymph nodes were subsequently ground with a syringe plunger under the microscope. The separated cells were then transferred to a sterile 15 ml tube and 6 × 10^6^ Sp2/0 cells were subsequently added to the mouse lymphocytes in 30 ml RPMI medium. The cell mixture was centrifuged at 150 × *g* for 10 min at room temperature. Cells were resuspended in 10 ml medium and 1.5 ml polyethylene glycol (PEG, Sigma-Aldrich) was added over 1 min and mixed with a Pasteur pipette. Successively 20 ml RPMI medium were added over 4 min and cells were centrifuged at 150 × *g* for 10 min and later resuspended in HAT medium containing Hyper and cultured for 7 days. After 7 days cell supernatant was screened for the presence of specific antibodies to the protein of interest by ELISA followed by Western blotting. Validated mother clones were subcloned by limited dilution to obtain monoclonal cell clones.

### Molecular cloning

The following plasmids were used for protein overexpression: pEGFP-C1, pcDNA 3.1(+), pCS2(+)-V5, pQCH6, pCS2(+)-FLAG, pcDNA5/FRT/TO with IRES2EGFP insert, and pET-30a(+). Cloning primers are listed in Table S2. All plasmids used in the manuscript were verified by sequencing. WT siRNA-resistant (R-) *SLFN5*, R-*SLFN5-SP* and R-*SLFN5-AP* constructs were synthesized by Invitrogen^TM^ Thermo Fisher Scientific^TM^ using GeneArt^TM^ Strings^TM^ DNA Fragments and optimized for *H. sapiens*. Mutation of codons in order to obtain siRNA-resistant gene sequence and to produce threonine-to-serine and threonine-to-alanine substitutions were designed taking into account the codon bias of *H. sapiens*. Sequences targeted by the RNAi oligos were mutated as follows: GCTCAAAGTGTCTATAGCT (SLFN5 RNAi #01), ACATATGGAGGCTCTCCTA (SLFN5 RNAi #02), TAAAGGGTACTCAATGATT (SLFN5 RNAi #03), CGAAGAATCCGACCTCTTG (SLFN5 RNAi #04). Codons coding for threonine residues were mutated to serine and alanine, respectively, as follows: TCT (T143S) and GCT (T143A), AGC (T355S) and GCG (T355A), TCT (T388S) and GCT (T388A), TCC (T449S) and GCC (T449A), TCC (T491S) and GCC (T491A), TCC (T522S) and GCC (T522A), and GCT (S786A).

### Flow cytometry

Cells were fixed by dropwise addition of 70% ice-cold reaction grade ethanol and stored at 4 °C for at least 1 h. Samples were subsequently stained with propidium iodide and RNase A for 1 h in the dark. Cell cycle analysis was performed using a Accuri C6 flow cytometer and analyzed with BD Accuri C6 software. Phospho-histone H3 (pHH3) staining was performed to discriminate cells in G_2_ and M. Before proceeding with PI staining, cells were fixed in a 0.25% Triton X-100 solution and incubated 15 min. on ice. Cells were resuspended in anti-phospho-histone H3 (ser10) antibody and incubated at room temperature for 2 h. Alexa Fluor 488 anti-rabbit IgG was used as secondary antibody. PI staining was then performed as previously described. Annexin V staining was performed using the Annexin V Apoptosis Detection Kit 1 (Becton Dickinson) according to manufacturer ´s protocol.

### Cell growth assays

The Sulforhodamine B (SRB) proliferation assay was used to assess cell viability. Medium was removed from the wells and cells were washed once with PBS and fixed in prechilled 10% trichloroacetic acid (TCA) (Sigma-Aldrich) for 30 min at 4 °C. Cells were subsequently washed two times with double-distilled water and stained with 0.4% SRB (Sigma-Aldrich) in 1% acetic acid for 20 min at room temperature protected from light. SRB was then removed, and cells were washed four times in 1% acetic acid. After complete light-protected drying, SRB was dissolved by addition of 10 mM Tris pH 8 and gentle shaking at room temperature for 2 h. A total of 100 μL of the solution were then transferred to a 96-well plate and absorbance at A510 was read with plate reader. Percentage of cell-growth inhibition was calculated in relation to a mock-treated control.

The IncuCyte® live-cell analysis system was additionally used to real-time track the growth of the cells subjected to gene knock-down and rescue.

### Immunofluorescence microscopy

For immunostaining, cells were fixed in 4% formaldehyde (FA) solution and permeabilized with 0.2% Triton X-100, followed by blocking for 1 h in 10% goat serum. Primary anti-SLFN5 #111/1, dialyzed against azide-free PBS, or anti-V5 (mouse monoclonal; Thermo Fisher Scientific^TM^) were diluted 1:500, respectively. Alexa Fluor 488 goat anti-mouse IgG (1:1000, Life Technologies) was used as a secondary antibody. DNA was counterstained with Hoechst 33342 (1:1000, Life Technologies^TM^). Images were acquired and analyzed using a Zeiss Cell Observer.Z1 system equipped with an AxioCam MRm camera or Axioskop equipped with an AxioCam MRm camera. Image analysis was performed with ZEN lite 2011 software.

### Live cell microscopy

Image acquisition was performed on a DeltaVision Elite system microscope (GE Healthcare), equipped with a CoolSNAP HQ2 camera (Photometrics), 40× oil immersion objective.

U2OS EGFP-PCNA stable cell line treated with siLuciferase (Control) or si*SLFN5* were cultured in eight-well Ibidi dishes (Ibidi). Before filming, the media was exchanged to Leibovitz’s L-15 (Life Technologies). Time-lapse videos were recorded every 7 min for 18–24 h. Subsequent data analysis was done using SoftWoRx (GE Healthcare), GraphPad Prism 9 (GraphPad Software, San Diego, California, USA) and Fiji.

### Immunoblotting

Harvested cells and *Xenopus* oocytes/embryos were lysed in lysis buffer (50 mM Tris/HCl, pH 7.4, 1 mM NaCl, 1 mM EDTA, 0.25% sodium deoxycholate, 1% NP-40) supplemented with protease and phosphatase inhibitor cocktail (Roche). If required, benzonase nuclease (Millipore Merk) was added at a concentration of 50 U/ml in order to dissolve DNA residues. Healthy tissue whole cell lysates were purchased from ProSci^TM^ Inc., supplied in SDS sample buffer containing 50 mM DTT. Lysates were resolved on 10% acrylamide gels following the Thomas and Kornberg protocol [23]. Biochemical protein fractionation assays were performed with the Subcellular Protein Fractionation Kit for Cultured Cells (Thermo Fisher Scientific^TM^) according to manufacturer’s protocol.

Following primary antibodies were used: anti-SLFN5 (mouse monoclonal; this manuscript #112/5/6; hybridoma culture supernatant 1:3), anti-SLFN12 (mouse monoclonal; this manuscript #78/2; hybridoma culture supernatant 1:3), anti-xslfn (mouse monoclonal; this manuscript #69/1; hybridoma culture supernatant 1:3), anti-SLFN11 (mouse monoclonal; Santa Cruz Biotechnology, 1:200), anti-β-actin-HRP (mouse monoclonal; Santa Cruz Biotechnology; 1:3000), anti-Cyclin B for human cells (mouse monoclonal; BD Biosciences, 1:2000), anti-Cyclin A (rabbit polyclonal, Santa Cruz Biotechnology, 1:2000), anti-Cyclin B2 for *X. laevis* (mouse monoclonal; Abcam; 1:500), anti-PP2A B Subunit (rabbit monoclonal; Cell Signaling Technology; 1:2000), anti-GFP (mouse monoclonal; Santa Cruz Biotechnology; 1:3000) anti-V5 (mouse monoclonal; 1:2000; Thermo Fisher Scientific), anti-Lamin B1 (mouse monoclonal; Santa Cruz Biotechnology; 1:1000), anti-TPX2 (rabbit monoclonal; Cell Signaling Technology; 1:2000), anti-H3K9me3 (rabbit monoclonal; Cell Signaling Technology; 1:2000), anti-GAPDH (mouse monoclonal; Santa Cruz Biotechnology; 1:2000), anti-FLAG (mouse monoclonal; Sigma-Aldrich; 1:4000).

### Immunoprecipitation

Proteins for co-immunoprecipitation (Co-IP) were isolated using nuclear extraction buffer (NEB; Tris-HCl 50 mM pH 8, EDTA 2 mM, NP-40 0.1%, Glycerol 10%) to extract nuclei from the cells and nuclear lysis buffer (NLB; Tris-HCl 25 mM pH 7.4, NaCl 150 mM, EDTA 1 mM, NP-40 1%, Glycerol 5%, Benzonase 1:100) to extract nuclear proteins. Both lysis buffers were supplemented with protein and phosphatase inhibitor cocktail (Roche).

Co-IP was performed under native conditions. A total of 1 mg protein was diluted in 500 μl co-IP buffer (CIB; Tris-HCl 25 mM pH 7.4, NaCl 150 mM, EDTA 1 mM, glycerol 5%, supplemented with protein and phosphatase inhibitor cocktail). For Co-IP the Pierce^TM^ Co-Immunoprecipitation (co-IP) Kit (Thermo Scientific^TM^) was used. Manufacturer’s protocol was adapted to the particular needs of the experiment: 50 μl resin slurry were washed twice with 200 μl 1× coupling buffer and 3 μg of anti-SLFN5 #112/5/6 antibody (dialyzed against azide-free PBS) diluted in 200 μl 1× coupling buffer were added to the resin. A total of 3 μl of sodium cyanoborohydride solution were added to every 200 μl reaction. The tubes rotated for 2 h at room temperature to allow antibody coupling to the resin. The resin was washed two times with 200 μl 1× coupling buffer and once with 200 μl quenching buffer. In total 3 μl of sodium cyanoborohydride solution were added to 200 μl quenching buffer and the solution was added to the resin and rotated 15 min at room temperature. The resin was subsequently washed twice with 200 μl 1× coupling buffer and finally 6 times with 150 μl wash buffer (WB; Tris-HCl 25 mM pH 7.4, NaCl 250 mM, EDTA 1 mM, glycerol 5%, supplemented with protein and phosphatase inhibitor cocktail). Resin was then washed once with 1× coupling buffer before proceeding with co-IP. 1 mg of protein lysate in 500 μl CIB was precleared for 1 h at 4 °C using 40 μl of control agarose resin slurry with gentle end-over-end mixing. Subsequently the lysate was rotated with IgG antibodies covalently immobilized to beads (IgG-control-IP) for 3 h at 4 °C. The supernatant was finally added to the anti-SLFN5-beads (Bait-IP) and rotated overnight at 4 °C. Bait-IP-beads were washed once with 200 μl CIB. Beads were then washed 6 times with WB and stored at −80 °C for mass spectrometry (MS) analysis or resuspended in 2× SDS sample loading buffer for polyacrylamide gel electrophoresis (PAGE) followed by immunoblotting. IgG-control beads pellet was washed and treated under the same conditions of bait-IP beads.

GFP-Trap (ChromoTek) was used to perform pulldown experiments of YFP-B55α. A total of 20 μl anti-GFP agarose affinity slurry was washed five times with PBS. Cell lysis was performed under the same conditions as for the SLFN5-Co-IP after transient overexpression of V5-SLFN5 fragments and YFP-B55α-induction for 12 h. A total of 50 μg protein lysate were diluted in 500 μl CIB and rotate overnight with 20 μl beads slurry. Subsequently beads were resuspended in 50 μl 2× SDS sample loading buffer for PAGE and SLFN5 recovery was assessed by immunoblotting using anti-V5 antibody.

### Mass spectrometry

Three biological replicates were performed per SLNF5 and IgG experiment. Beads were resuspended in 50 mM ammonium bicarbonate (NH_4_HCO_3_). DTT was added to 7 mM final concentration and samples were subjected to reduction by heating at 55 °C for 30 min, followed by disulfide bond alkylation with 12 mM iodoacetamide at room temperature for 40 min in the dark. Reaction was quenched with DTT and proteins were trypsin digested on beads overnight at 37 °C. Digested peptides were lyophilized and desalted on C18 stage tips [24]. Samples were loaded in 1% TFA and 5% acetonitrile and eluted with high organic solvent (80% acetonitrile). Eluted peptides were lyophilized, resuspended in 0.1% trifluoroacetic acid (TFA) in water and subjected to LC-MS/MS analysis.

Peptides were injected and analyzed on an Q Exactive^TM^-HF (Thermo Scientific) coupled to a Thermo EASY LC 1200 UPLC system. Samples were loaded onto trap column (Acclaim^TM^ PepMap^TM^ 100 C18 Nano-Trap 2 cm × 100 μm × 5 μm) with Buffer A (0.1% formic acid in water) and separated over a 50 cm analytical column (Acclaim^TM^ PepMap^TM^ RSLC, 75 μm × 2 μm) using a 105 min linear gradient from 3% to 40% Buffer B (0.1% formic acid, acetonitrile 80% in water). MS2 fragmentation was set to HCD.

RAW data were processed with Maxquant using default settings [25]. MSMS spectra were searched against the *H. sapiens* Uniprot database concatenated to a database containing protein sequences of contaminants. Enzyme specificity was set to trypsin/P, allowing a maximum of two missed cleavages. Cysteine carbamidomethylation was set as fixed modification, while methionine oxidation and protein N-terminal acetylation were used as variable modifications. Global false discovery rate for both protein and peptides was set to 1%. The match-between-runs option was enabled. Intensity-based quantification options (iBAQ and LFQ) were calculated. Data analysis was performed with Perseus software [26]. Upon filtering for contaminants and reverse, only proteins with at least 1 unique peptide in all the three target biological replicates were considered for further analysis. For the proteins identified, the ratio between the LFQ value in the target over the respective control was calculated and only proteins with LFQ ratio greater than 2 were in all the three biological replicates were used for table generation. Imputation was applied for proteins without an LFQ value in the IgG negative control were the LFQ ratio was set to 15 as highest value.

### In vitro kinase and phosphatase assay

In vitro phosphatase assays were performed with PP2A-B55a, purified from HeLa cell extracts as described previously [14]. 15 µg of recombinant 6×His-SLFN5-DBD protein was incubated at 30 °C for 60 min with the Cyclin B-CDK1 complex (Sigma Aldrich) in a 50 µL reaction in kinase buffer (50 mM Tris–HCl pH 7.5, 10 mM MgCl_2_, 0.1 mM EDTA, 2 mM DTT, 0.01% Brij35) with 500 µM ATP and 1 µCi (𝛄-^32^P)-ATP (PerkinElmer) at 30 °C for 60 min. Reactions were stopped by the addition of 10 µM RO-3306 (Calbiochem). PD Spin Trap G25 columns (GE Healthcare) were used to exchange the buffer to phosphatase buffer (50 mM Tris pH 7.4,1 mM MnCl_2_, 1 mM DTT, 0.1% IGEPAL, 150 mM NaCl) and non-sticky tubes were pre-treated with blocking buffer (50 mM Tris pH 7.4, 0.1 mM MnCl_2_, 1 mM MgCl_2_, 1 mM DTT, 0.1% NP40, 300 mM NaCl, 2 mg/ml BSA) on ice for the dephosphorylation reactions. 10 µL PP2A-B55α holoenzyme was added to 84 µL of phosphorylated substrate Samples were taken out at the indicated time-points, added to 4×SDS loading buffer and boiled for 7 min at 95 °C. Samples were separated by SDS–PAGE. Gels were dried, exposed for 3 days, and imaged on Typhoon FL 950 (GE Healthcare). Colloidal blue staining was performed using the Colloidal Blue Staining Kit (Thermo Fisher Scientific^TM^), according to the manufacturer ´s instructions.

### Protein expression in *E. coli*

SLFN5-DBD was expressed in BL21(DE3) cells at 18 °C for 12 h following induction. The cells were pelleted and resuspended in buffer L (50 mM NaP pH = 7.5, 300 mM NaCl, 10 mM imidazole, 10% glycerol, 0.5 mM TCEP, benzonase, protease inhibitors) and lysed by sonication. Following clarification of extract by centrifugation the sample was applied to a 5 ml HiTrap Ni column and washed with buffer L (without benzonase and protease inhibitors). The protein was eluted from the column using a gradient of imidazole (10–500 mM) and peak fractions applied to a Superdex 200 16/60 column equilibrated with buffer G (50 mM NaP pH = 7.5, 150 mM NaCl, 10% glycerol, 0.5 mM TCEP). Peak fractions were pooled and analysed by SDS-PAGE and confirmed by mass spectrometry.

### Collection and injection of *X. laevis* oocytes

*X. laevis* oocytes were harvested from unprimed *Xenopus* females. Ovaries were collected and enzymatically digested using collagenase D (Roche). Stage VI oocytes were selected according to the classification of Dumont [27] and recovered in oocyte medium (5 mM HEPES, 82.5 mM NaCl, 2.5 mM KCl, 1 mM CaCl_2_, 1 mM MgCl_2_, 3.8 mM NaOH, 1 mM Na_2_HPO_4_, pH 7.8) [28] overnight at 18 °C. Oocytes were then injected with an antisense Morpholino (purchased from Gene Tools, LLC, Philomat, Oregon, USA) targeting the 5′ UTR of *xslfn* with 40 or 80 ng (Table S3). The control group was injected with 80 ng of standard control Morpholino (Table S3). Morpholino designs were BLASTed to check for possible cross-identification of other gene sequences. After 24 h of injection, the oocytes were treated with 10 µg/ml progesterone (Sigma-Aldrich) to induce GVBD. The appearance of germinal vesical breakdown (GVBD) was exploited as a marker of maturation (CSF-arrest) and was performed in a blinded manner. For microinjection a Pico-Injector PLI-100, Harvard Instruments, was used. In vitro mRNA synthesis for rescue experiments was performed using the MEGAscript^TM^ SP6 Transcription Kit (Thermo Fisher Scientific^TM^), according to the manufacturer ´s protocol. A total of 5 ng mRNA coding for full-length FLAG-tagged *xslfn* were injected through the animal pole in the cytoplasm.

### Fertilization and collection of *X. laevis* embryos

Females of *X. laevis* were primed with 600 U of human chorionic gonadotropin (hCG, Genaxxon) into the dorsal lymph sac. After 24 h the frogs were gently squeezed to harvest eggs which were transferred to a Petri dish for fertilization. A testis from an adult *Xenopus* male was shred mazerated with scissors and forceps to release sperms which were then carefully pipetted on eggs, incubated for 3 min, flooded with 0.1× Barth (8.8 mM NaCl, 0.1 mM KCl, 0.24 mM NaHCO_3_, 0.082 mM MgSO4·7H_2_O, 0.033 mM Ca(NO_3_)2·4H_2_O, 0.041 mM CaCl_2_·2H_2_O, 1 mM HEPES (pH7.6)) and incubated for 15 min until appearance of cortical rotation which was used as marker of successful fertilization. Embryos were subsequently dejellied using 2% cysteine (Sigma-Aldrich) dissolved in water and the pH adjusted with NaOH to 7.8–8.0. After dejellying, embryos were washed three times in 0.1× Barth, three times in 0.3× Barth, transferred to a Petri dish and cultured at 14–18 °C in 0.1× Barth solution. After reaching the desired stage, five embryos were pooled per sample, flash frozen in liquid nitrogen and stored at -80 °C until use. Dephosphorylation of *X. laevis* protein lysates was performed using λ-protein phosphatase (New England BioLabs^®^).

#### Quantification and statistical analysis

GraphPad Prism 7.0 (GraphPad software, San Diego, CA) was used for all statistical analysis. One-way analysis of variance (ANOVA) was used to determine statistical significance. Data are represented as mean ± standard deviation. Data were considered statistically significant with the following *p*-values: *P ≤ 0.05, **P ≤ 0.01, ***P ≤ 0.001, ****P ≤ 0.0001, ns, no significant difference.

## Supplementary information

Supplementary figures 1-4 with legends

Supplementary tables

Control movie

SLFN5 RNAi movie

Supplemental excel files

Supplementary Mass Spec data

Original Data File

Quantification of SLFN5 oscillations

## Data Availability

All data supporting the findings of this study are available within the article or its supplementary materials (uncropped images from Western blots, supplementary tables with oligonucleotides, supplementary excel files with Mass Spectrometry raw data and its analysis).
