# Supplementary material for: Human SLFN5 and its Xenopus Laevis ortholog regulate entry into mitosis and oocyte meiotic resumption
Source: Cell Death Discov. 2022 Dec 8;8:484. doi: 10.1038/s41420-022-01274-0 (PMC9729291; doi:10.1038/s41420-022-01274-0)
Supplement: Supplementary file 1 — Supplementary figures 1-4 with legends [file 41420_2022_1274_MOESM1_ESM.docx]

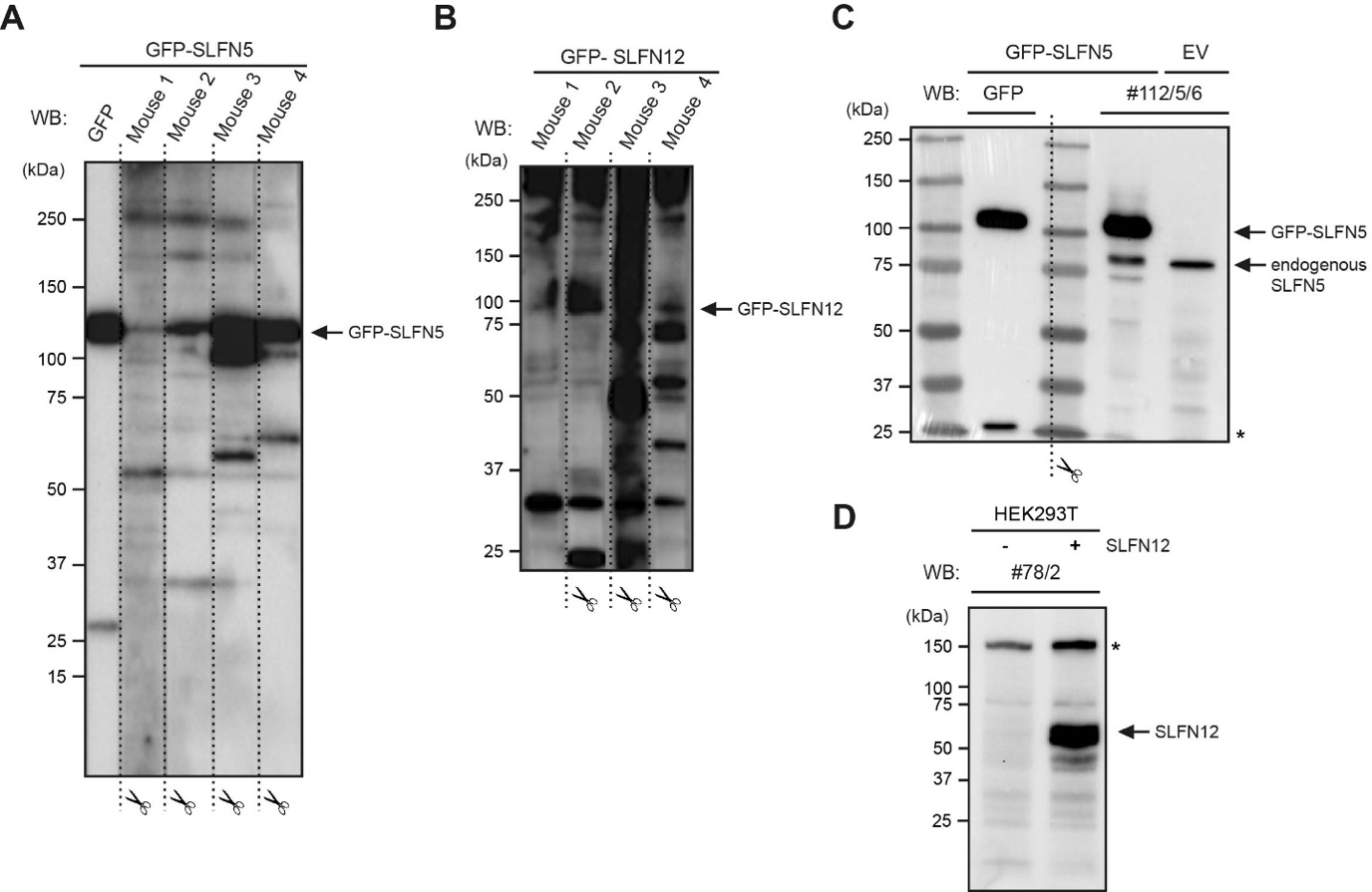


**Supplementary Figure 1**


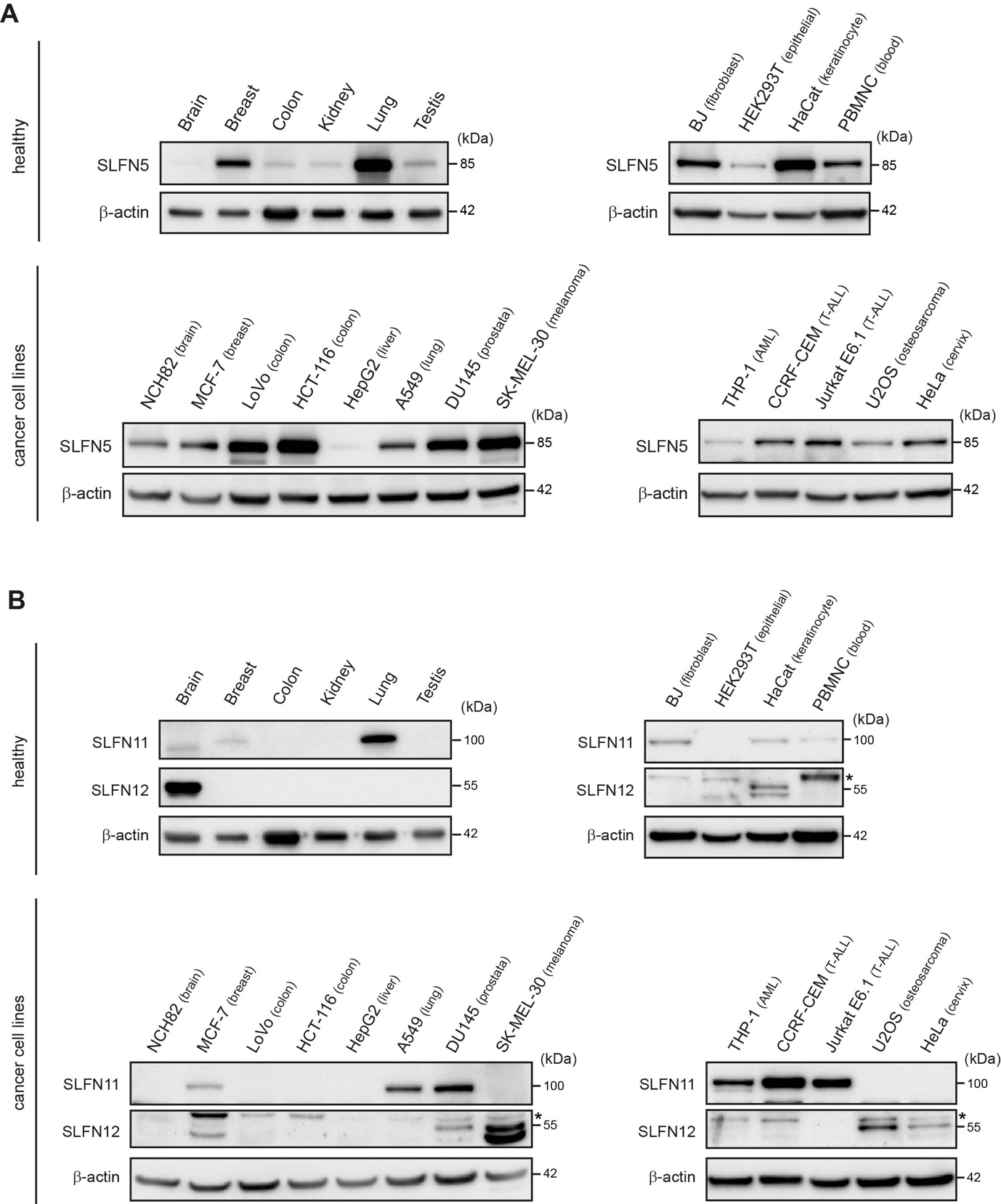


**Supplementary Figure 2**


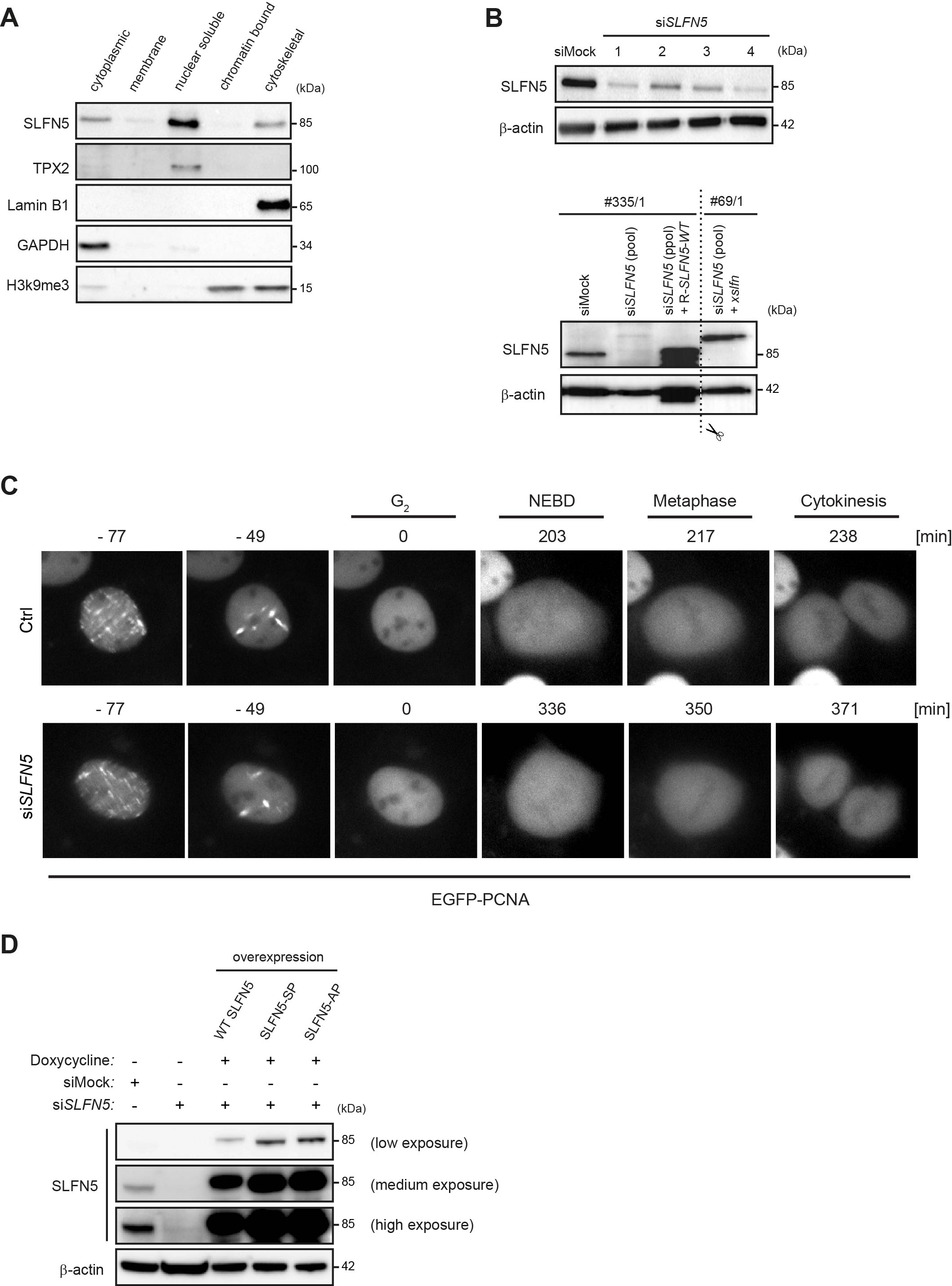


**Supplementary Figure 3**


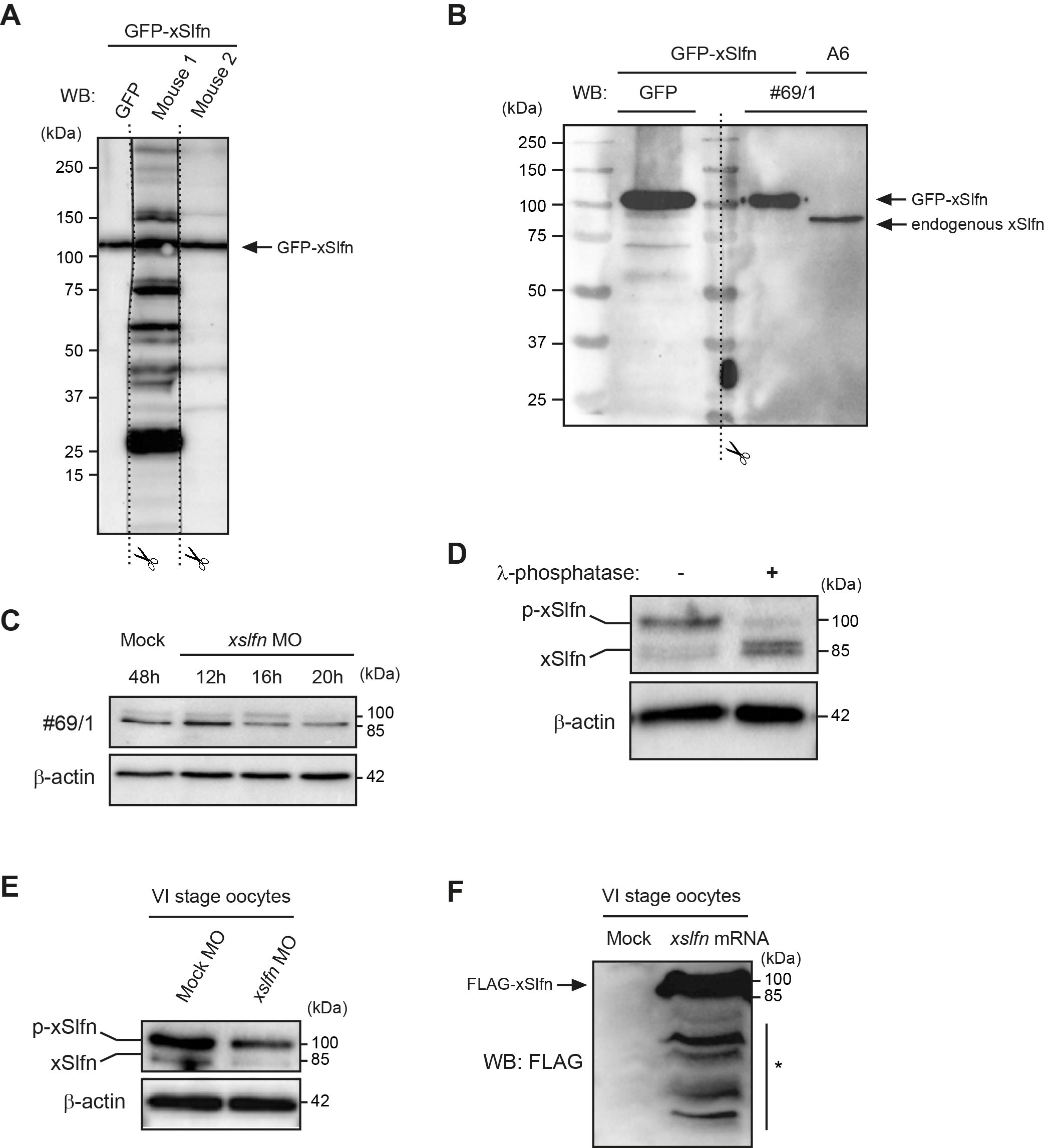


**Supplementary Figure 4**

**SUPPLEMENTARY FIGURE LEGENDS**

**Figure S1. Characterization of a novel antibody against human SLFN5 and SLFN12.**

**A-B)** Mouse anti-sera towards SLFN5 and SLFN12. Protein lysates from HEK293T cells transiently transfected with GFP-SLFN5 or GFP-SLFN12 were used. Membranes were cut in slices and probed with four different mouse anti-sera. An anti-GFP antibody was used as positive control. **C-D)** Assessment of subclone specificity with protein lysates from HEK293T cells transiently transfected with GFP-SLFN5 (**C**) or untagged SLFN12 (**D**) as well as with protein lysates from Mock-transfected HEK293T cells. Membranes were probed with anti-GFP antibody (as positive control) and anti-SLFN5 antibody (clone #112/5/6) (**C**), or anti-SLFN12 antibody (clone #78/2) (**D**), respectively. EV, empty vector. Asterisks indicate nonspecific bands.

**Figure S2. SLFN5, SLFN11 and SLFN12 protein expression analysis in healthy tissues and cancer cell lines.**

**A-B)** Western blotting of SLFN5 (A), and SLFN11 and SLFN12 (B) protein expression. Whole cell lysates from healthy human primary tissue samples as well as non-transformed and cancer cell lines from different tissue types were immunoblotted for SLFN5 (#112/5/6), SLFN11 and SLFN12. β-actin was used as loading a control. Asterisks indicate nonspecific bands. PBMNC, peripheral blood mononuclear cells. T-ALL, T-cell acute lymphoblastic leukemia; AML, acute myeloid leukemia.

**Figure S3. Biochemical fractionation assay, RNAi specificity against *SLFN5*, still images of Live cell microscopy related to Figure 1F-G and controls related to Figure 3B-C.**

**A)** Subcellular protein fractionation of U2OS cells confirms SLFN5 nuclear localization. Immunoblotting for proteins from different cellular compartments was used to show low contamination among fractions. **B)** siRNA-mediated SLFN5 depletion with four different antisense oligomers confirms antibody specificity for human SLFN5 (upper blot). siRNA-mediated SLFN5 depletion with a pool of four oligos and SLFN5 and xSLFN overexpression (lower blot). β-actin was used as a loading control. **C)** Representative stills of U2OS EGFP-PCNA stable cell line treated with siLuciferase (Ctrl) or si*SLFN5*. G2-to-NEBD (Nuclear envelope breakdown) takes on average 200 min in Ctrl and 360 min in si*SLFN5*. NEBD-to-Cytokinesis takes on average 35 min in both conditions (for details please refer to main Figure 1F-G and text). **D)** Downregulation of SLFN5 Doxycycline-induced overexpression of WT SLFN5, SLFN5-SP and SLFN5-AP assessed by Western blotting.

**Figure S4. Characterization of a novel antibody against *Xenopus laevis* Slfn (xSlfn) and *xslfn* Morpholino titration.**

**A)** Mouse anti-sera towards xSlfn. Protein lysate from HEK293T cells transiently transfected with GFP-xSlfn was used. Membranes were cut in slices and probed with two different mouse anti-sera. An anti-GFP antibody was used as positive control. **B)** Assessment of subclone #69/1 specificity with protein lysates from HEK293T cells transiently transfected with GFP-SLFN5 and non-transfected *Xenopus* A6 kidney normal cells. **C)** Subclone #69/1 specificity was confirmed by Morpholino-mediated xSlfn depletion in *X. laevis* embryos. **D)** xSlfn post-translational phosphorylation is removed by λ-phosphatase treatment of whole-embryo crude protein extracts. **E)** Morpholino-mediated xSlfn depletion in oocytes was confirmed by Western blotting with anti-xSlfn antibody 24 h after Morpholino transfection. Oocytes were sampled from the experiment depicted in Figure 4B. **F)** FLAG-xSlfn protein overexpression after FLAG-*xslfn* mRNA microinjection was confirmed by Western blotting with an anti-xSlfn antibody. Oocytes were sampled from the experiment shown in Figure 4B. Asterisks indicate nonspecific bands. **C-D** and **E**) β-actin was used as a loading control.
