# Supplementary material for: Human SLFN5 and its Xenopus Laevis ortholog regulate entry into mitosis and oocyte meiotic resumption
Source: Cell Death Discov. 2022 Dec 8;8:484. doi: 10.1038/s41420-022-01274-0 (PMC9729291; doi:10.1038/s41420-022-01274-0)
Supplement: Supplementary file 2 — Supplementary tables [file 41420_2022_1274_MOESM2_ESM.docx]

| **REAGENT or RESOURCE** | **SOURCE** | **IDENTIFIER** |
| --- | --- | --- |
| RNAi Oligonucleotides | | |
| siRNA SLFN5: 5’-GCACAGAGCGUUUACAGUU-3’ | GE Healthcare Dharmacon™ | Cat#D-027164-01 |
| siRNA SLFN5: 5’-GCACAUGGAAGCCCUGUUA-3’ | GE Healthcare Dharmacon™ | Cat#D-027164-02 |
| siRNA SLFN5: 5’-CAAGGGCUAUUCUAUGAUA-3’ | GE Healthcare Dharmacon™ | Cat#D-027164-03 |
| siRNA SLFN5: 5’-UGAGGAGUCUGAUCUGUUA-3’ | GE Healthcare Dharmacon™ | Cat#D-027164-04 |
| siRNA PPP2R2A: 5’-GAAAUUACAGACAGGAGUU-3’ | GE Healthcare Dharmacon™ | Cat#MU-004824-01 |
| siRNA PPP2R2A: 5’-UAUCAAGCCUGCCAAUAUG-3’ | GE Healthcare Dharmacon™ | Cat#MU-004824-02 |
| siRNA PPP2R2A: 5’-UAUGAUGACUAGAGACUAU-3’ | GE Healthcare Dharmacon™ | Cat#MU-004824-03 |
| siRNA PPP2R2A: 5’-GCAGAUGAUUUGCGGAUUA-3’ | GE Healthcare Dharmacon™ | Cat#MU-004824-04 |
| siRNA PPP2CA: 5’-AACCUUAAGAGCUACAAGCAG-3’ | Ambion^®^, Life Technologies | Cat#4390824  siRNA ID#s10958 |
| siRNA PPP2CB: 5’-ACAACGAUAACAGUAAUUGGG-3’ | Ambion^®^, Life Technologies | Cat#4390824  siRNA ID#s10961 |
| siRNA Non-Targeting Pool #2:  5’-UAAGGCUAUGAAGAGAUAC-3’;  5’-AUGUAUUGGCCUGUAUUAG-3’;  5’-AUGAACGUGAAUUGCUCAA-3’;  5’-UGGUUUACAUGUCGACUAA-3’ | GE Healthcare Dharmacon™ | Cat#D-001206-14 |

**Supplementary Table S1**

| **REAGENT or RESOURCE** | **SOURCE** | **IDENTIFIER** |
| --- | --- | --- |
| Cloning Primers | | |
| *SLFN5* full-length (pEGFP-C1; BspEI) forward:  5’-ATGCCATGCTATGCCGGAATGAGTCTTAGG-3’ | Eurofins Genomics | N/A |
| *SLFN5* full-length (pEGFP-C1; HindIII) reverse:  5’-GCATGCATGCATAAGGTTTCACACAGAAGCCTT-3’ | Eurofins Genomics | N/A |
| *SLFN12* full-length (pEGFP-C1; BspEI) forward:  5’-ATGCCATGCTATGAACATCAGTGT-3’ | Eurofins Genomics | N/A |
| *SLFN12* full-length (pEGFP-C1; HindIII) reverse:  5’-GCATGCATGCGGTGAGCCTTCGA-3’ | Eurofins Genomics | N/A |
| *SLFN5* full-length (pcDNA3.1(+); NheI) forward:  5’-ATGCGCTAGCATGAGTCTTAGGA-3’ | Eurofins Genomics | N/A |
| *SLFN5* full-length, *SLFN5^helicase^* (pcDNA3.1(+), pCS2(+)-V5; XhoI) reverse:  5’-GCATCTCGAGCACAGAAGCCTT-3’ | Eurofins Genomics | N/A |
| *SLFN12* full-length (pcDNA3.1(+); NheI) forward:  5’-ATGCGCTAGCATGAACATCAGTGT-3’ | Eurofins Genomics | N/A |
| *SLFN12* full-length (pcDNA3.1(+); XhoI) reverse:  5’-GCATCTCGAGGGTGAGCCTTCGA-3’ | Eurofins Genomics | N/A |
| *SLFN5* full-length, *SLFN5^DBD^* (pCS2(+)-V5; BamHI) forward:  5’-ATGCGGATCCATGAGTCTTAGGATTGATGTGGATA-3’ | Eurofins Genomics | N/A |
| *SLFN5^DBD^* (pCS2(+)-V5; XhoI) reverse:  5’-GCATCTCGAGTCATACTGGAAAGTAGCGTTTCTG-3’ | Eurofins Genomics | N/A |
| *SLFN5^NTPase^* (pCS2(+)-V5; BamHI) forward:  5’-ATGCGGATCCTTTTCAGACAGAGTGGTATA-3’ | Eurofins Genomics | N/A |
| *SLFN5^NTPase^* (pCS2(+)-V5; XhoI) reverse:  5’-GCATCTCGAGTCAGTCCCCATCTTCAGTACGGAA-3’ | Eurofins Genomics | N/A |
| *SLFN5^helicase^* (pCS2(+)-V5; BamHI) forward:  5’-ATGCGGATCCTGGTATGGGAAAGCAAAGTTC-3’ | Eurofins Genomics | N/A |
| xSlfn (pEGFP-C1; BspEI) forward:  5’-ATGCCATGCTATGATGGAAGATTCTCTTTCCAGTG-3’ | Eurofins Genomics | N/A |
| xSlfn (pEGFP-C1; HindIII) reverse:  5’-GCATGCATGCTTAAATTTTAAAATATTTTCTTCGCACAAA-3’ | Eurofins Genomics | N/A |
| xSlfn (pcDNA3.1(+); NheI) forward:  5’-ATGCGCTAGCATGGAAGATTCTCTTTCCAGTG-3’ | Eurofins Genomics | N/A |
| xSlfn (pcDNA3.1(+); XhoI) reverse:  5’-GCATCTCGAGTTAAATTTTAAAATATTTTCTTCGCACAAA-3’ | Eurofins Genomics | N/A |
| xSlfn (pCS2(+)-FLAG; BamHI) forward:  5’-ATGCGGATCCATGGAAGATTCTCTTTCCAGTG-3’ | Eurofins Genomics | N/A |
| xSlfn (pCS2(+)-FLAG; XhoI) reverse:  5’-GCATCTCGAGTTAAATTTTAAAATATTTTCTTCGCACAAA-3’ | Eurofins Genomics | N/A |
| *SLFN5^C-ter^* (pQCH6; BamHI) forward:  5’- GGCGGATCCATGGAACGTCATGGAGTAGGAT-3’ | Eurofins Genomics | N/A |
| *SLFN5^C-ter^* (pQCH6; HindIII) reverse:  5’- GCCAAGCTTTCTGTCTTTAACACAGTGTG-3’ | Eurofins Genomics | N/A |

**Supplementary Table S2**

| **REAGENT or RESOURCE** | **SOURCE** | **IDENTIFIER** |
| --- | --- | --- |
| Morpholino Oligomers | | |
| *xslfn* 5’-UTR Morpholino:  5’-ATGAAAACTATTCCTGTCTGTCCAG-3’ | Gene Tools, LLC, Philomat, Oregon, USA | N/A |
| *X. laevis* Standard Control Morpholino:  5’- CCTCTTACCTCAGTTACAATTTATA-3’ | Gene Tools, LLC, Philomat, Oregon, USA | N/A |

**Supplementary Table S3**
