# Supplementary material for: Human SLFN5 and its Xenopus Laevis ortholog regulate entry into mitosis and oocyte meiotic resumption
Source: Cell Death Discov. 2022 Dec 8;8:484. doi: 10.1038/s41420-022-01274-0 (PMC9729291; doi:10.1038/s41420-022-01274-0)

Figure 1H

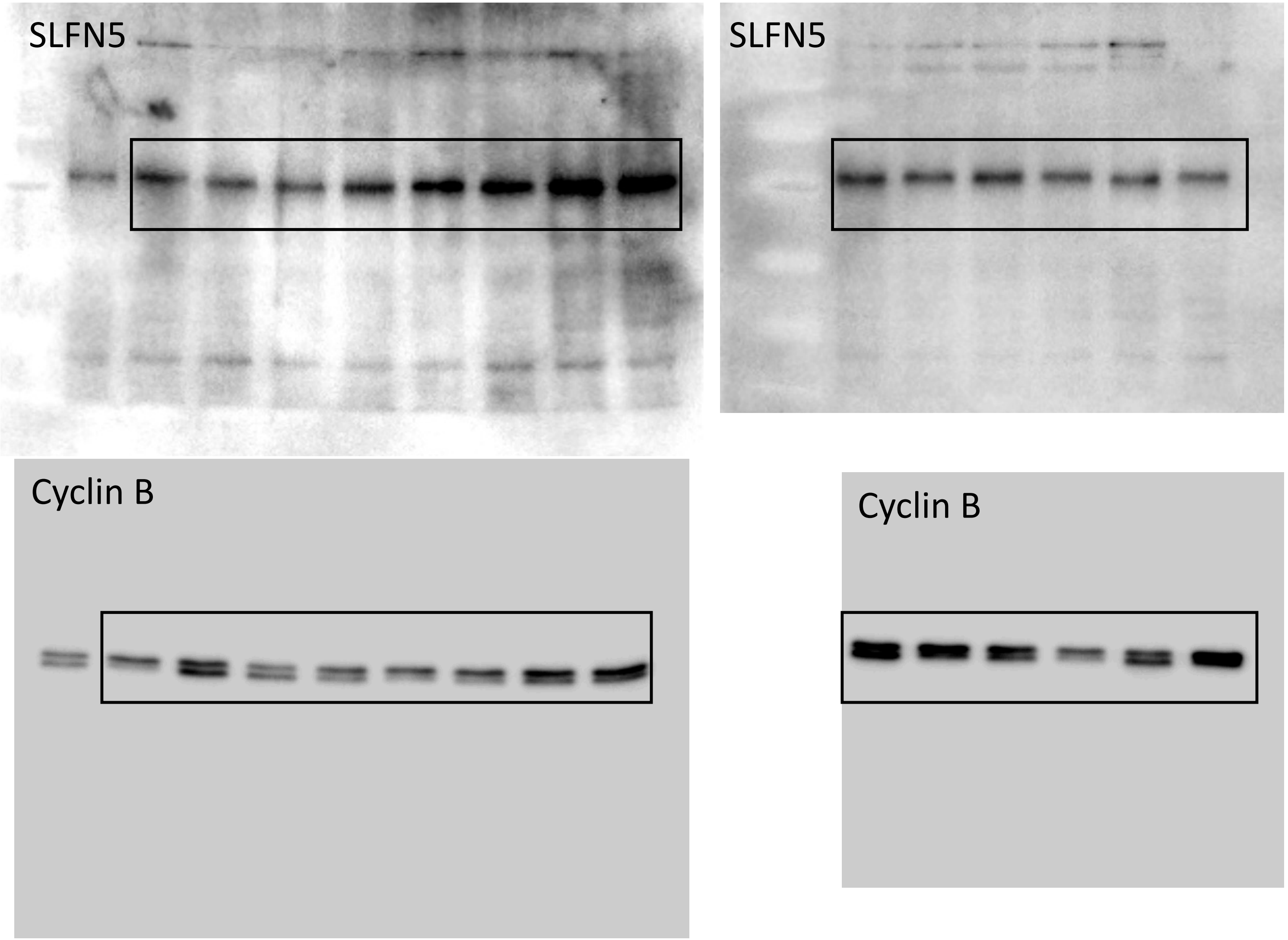

Figure 1H

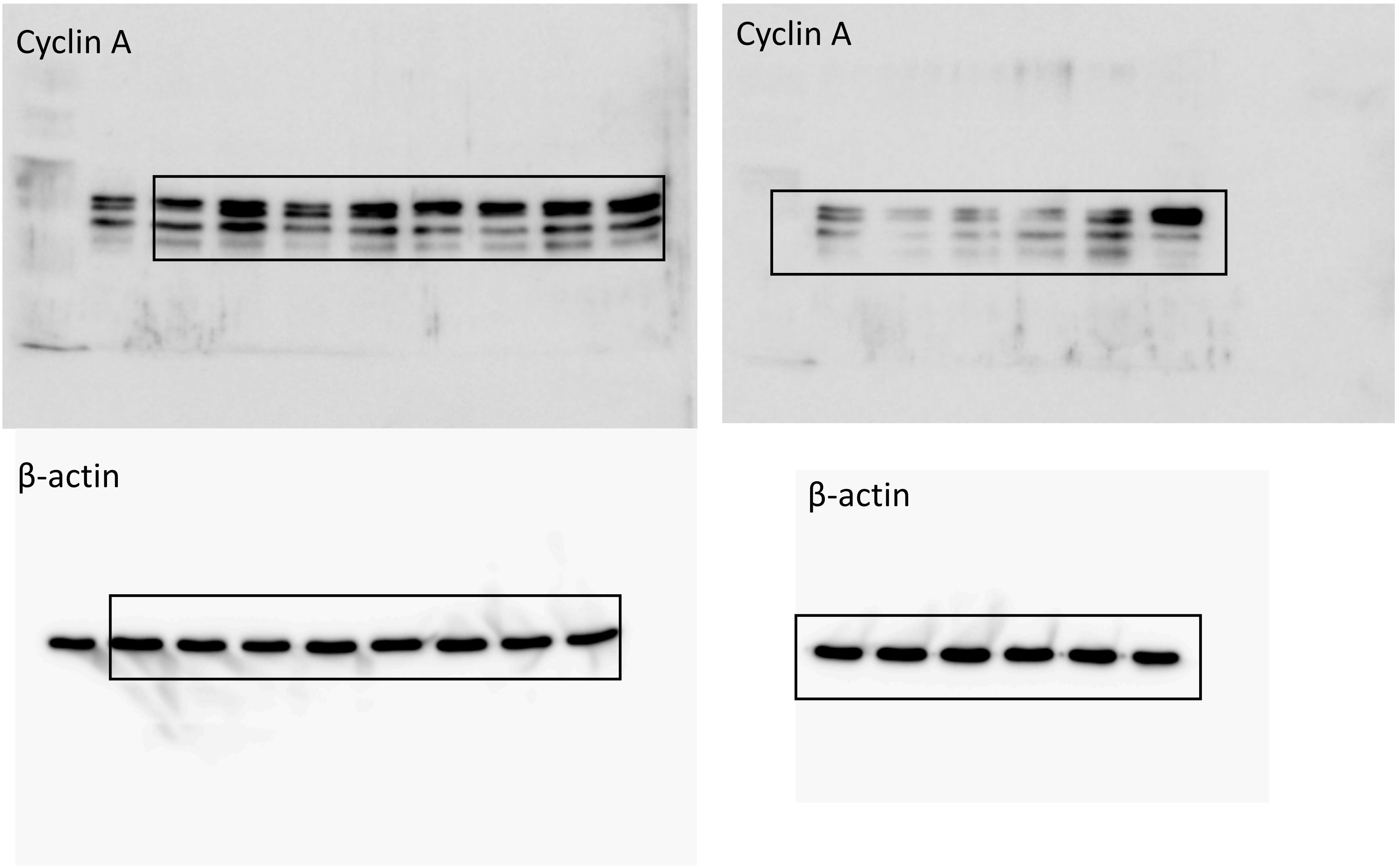

Figure 2B

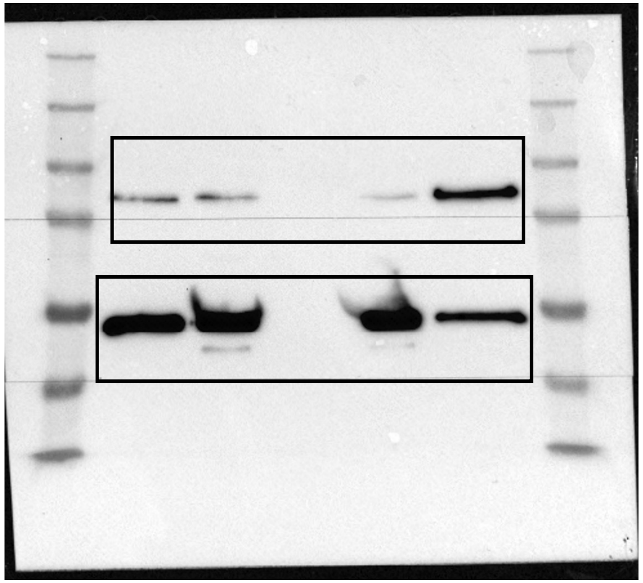

Figure 2C

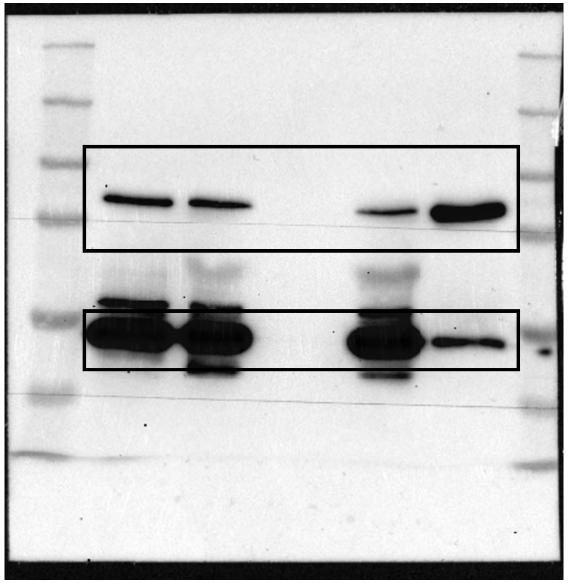

Figure 2E

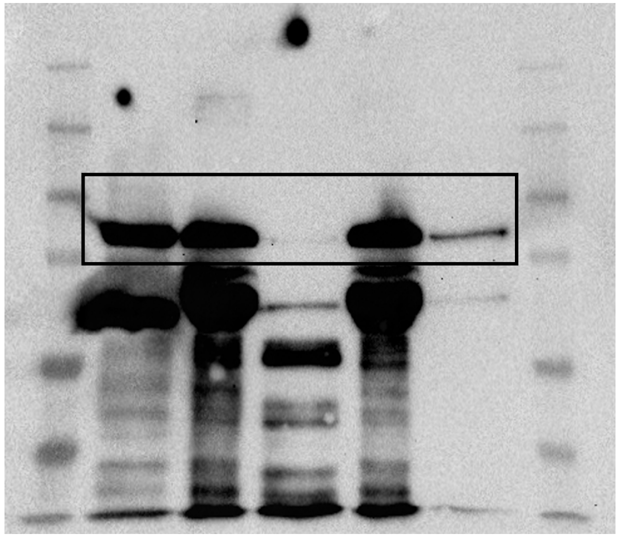

Figure 3E

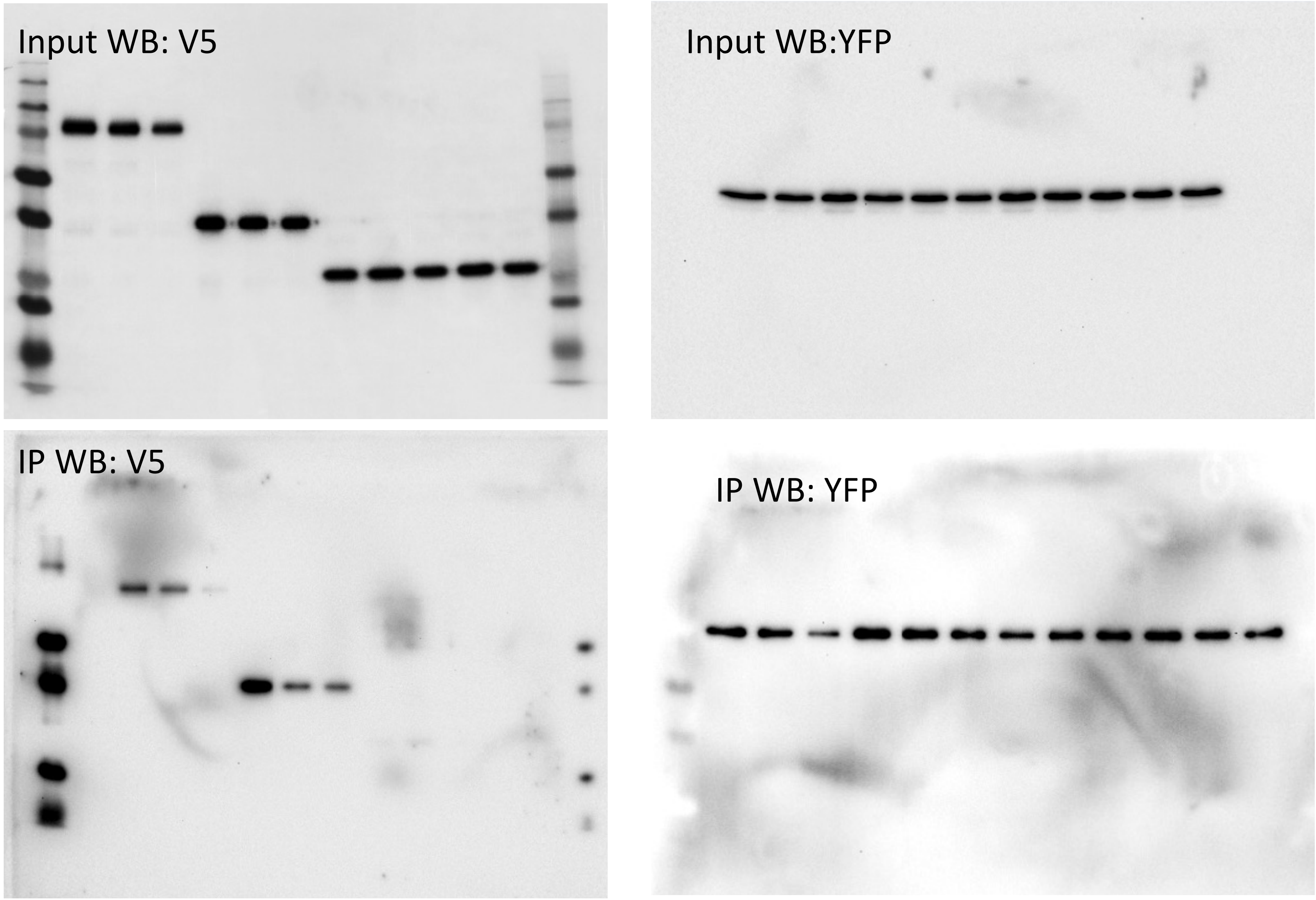

Figure 3F

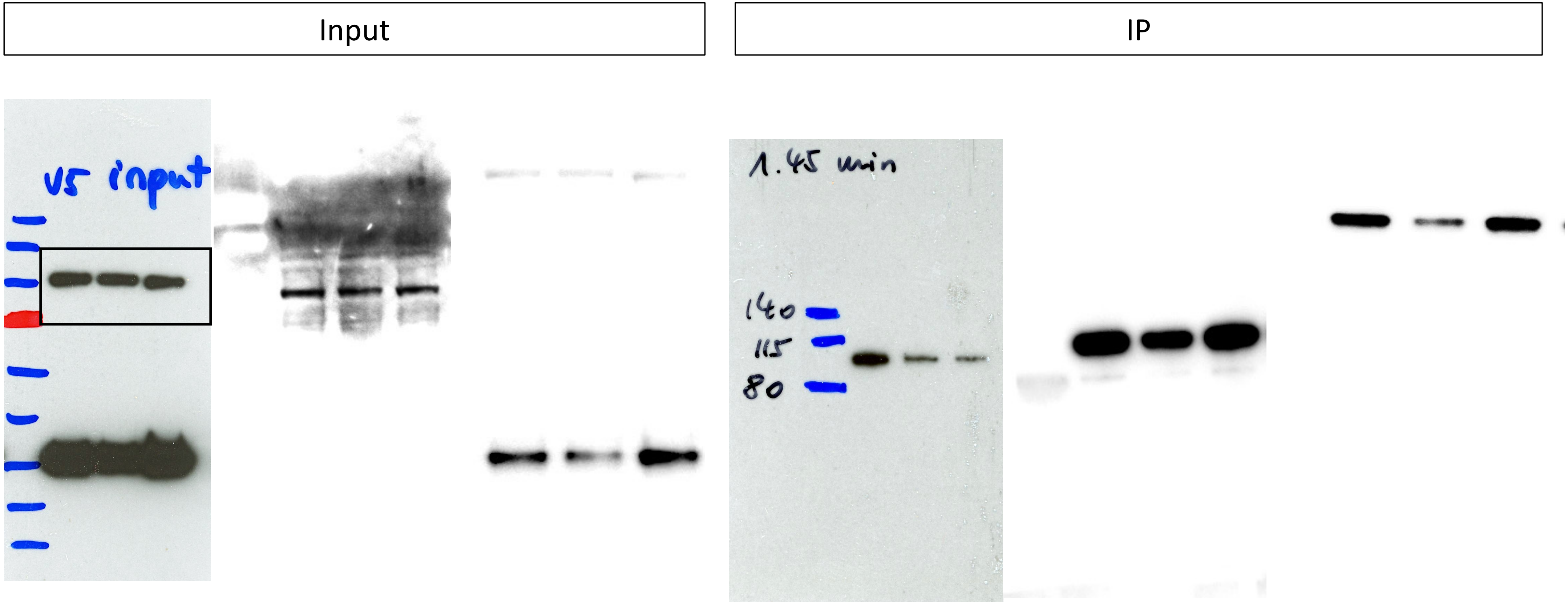

Figure 4A

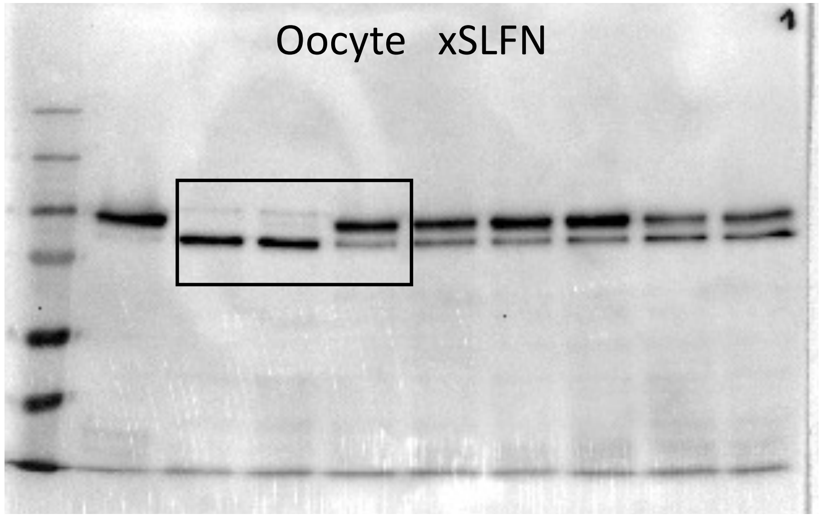

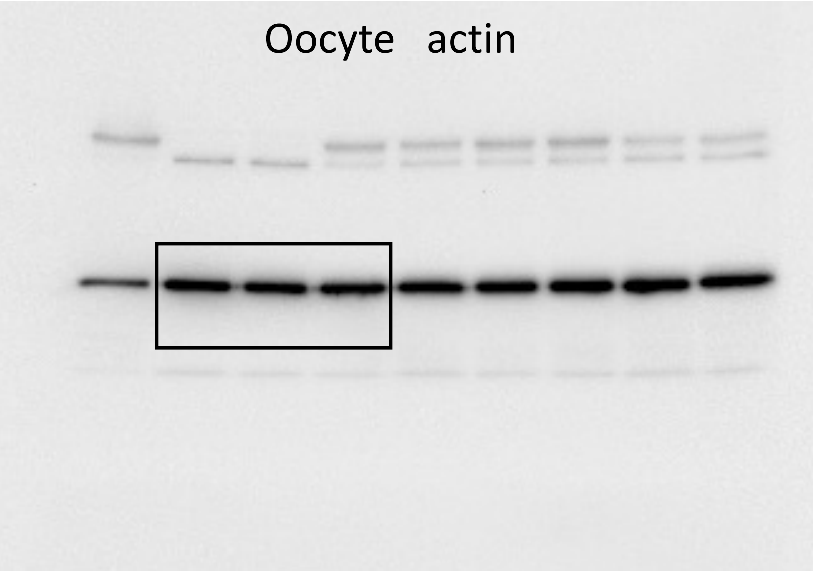

Figure 4A

Meiosis II to 2nd cleavage: xSLFN (PVDF membrane)

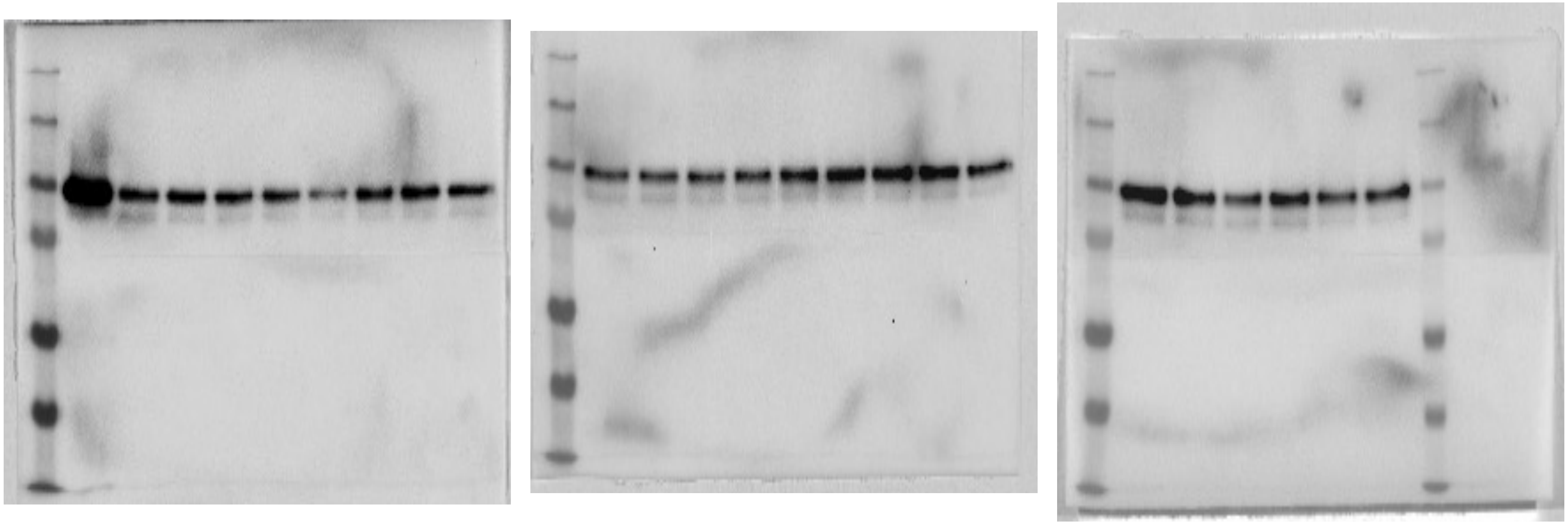

Figure 4A

Meiosis II to 2nd cleavage: xCyclin B2 (Nitrocellulose membrane)

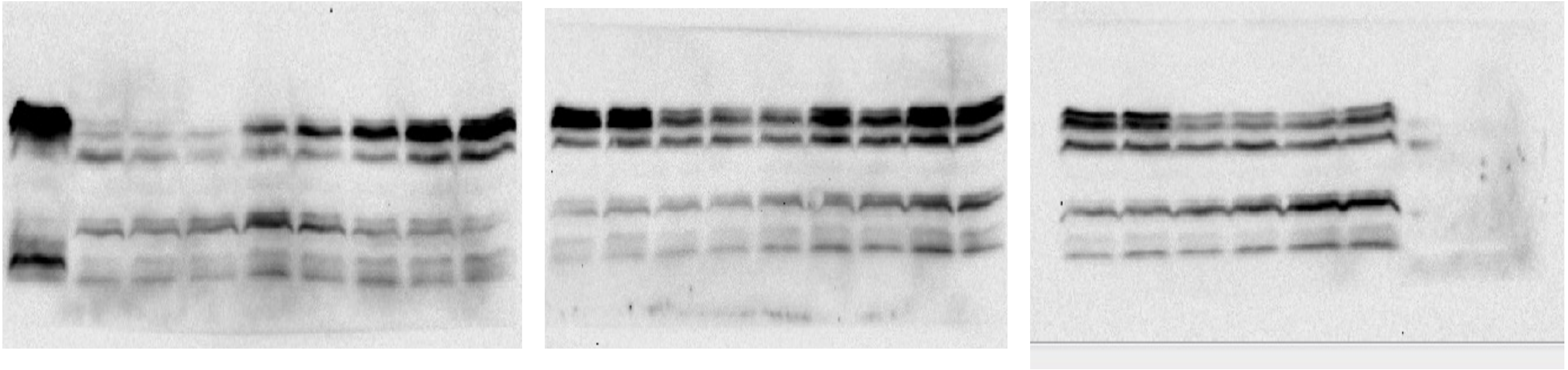

Figure 4A

Meiosis II to 2nd cleavage: actin (PVDF membrane)

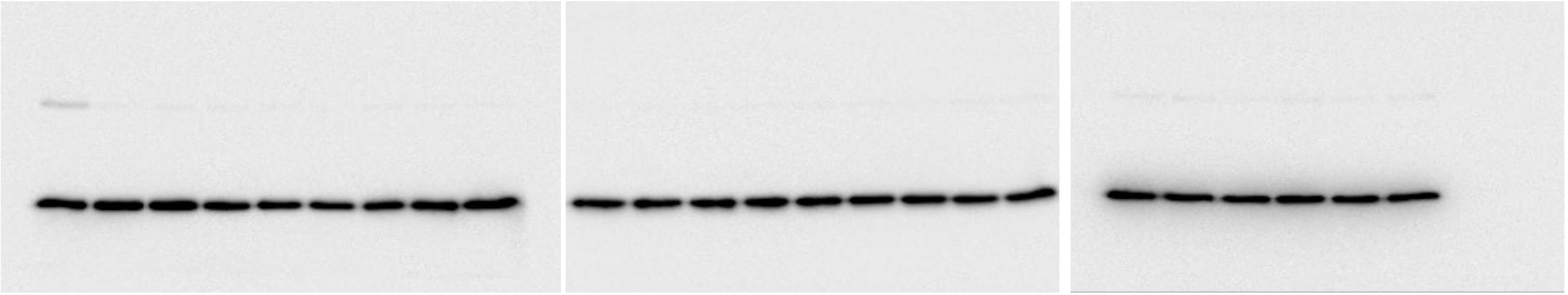

Supplementary Figure 1

A

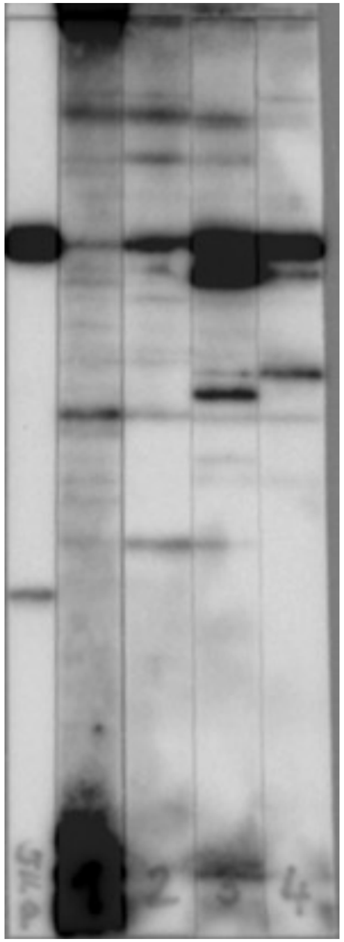

B

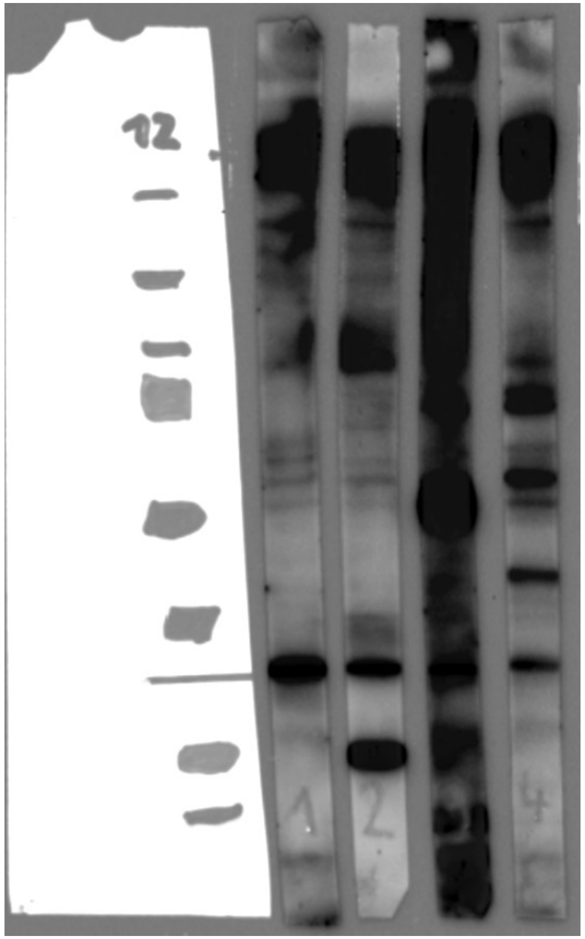

Supplementary Figure S1

C

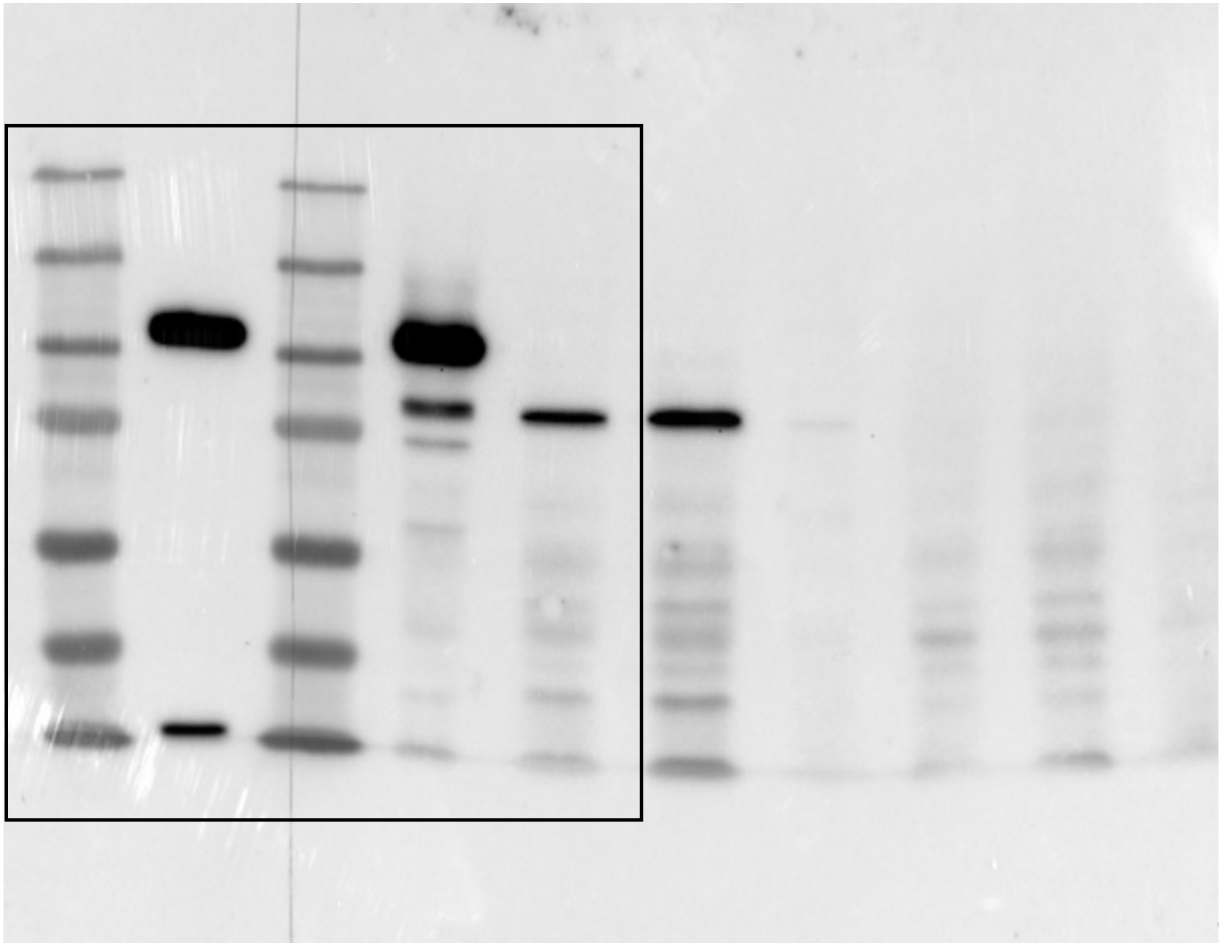

D

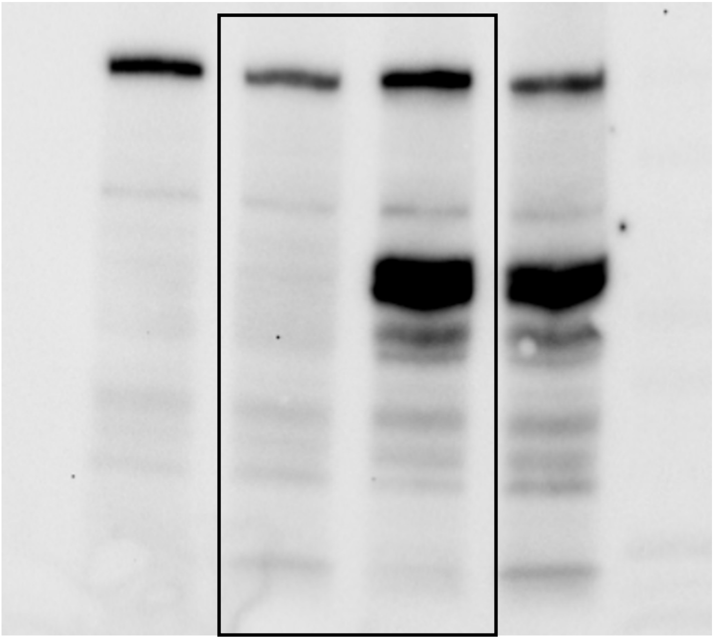

Supplementary Figure S3A

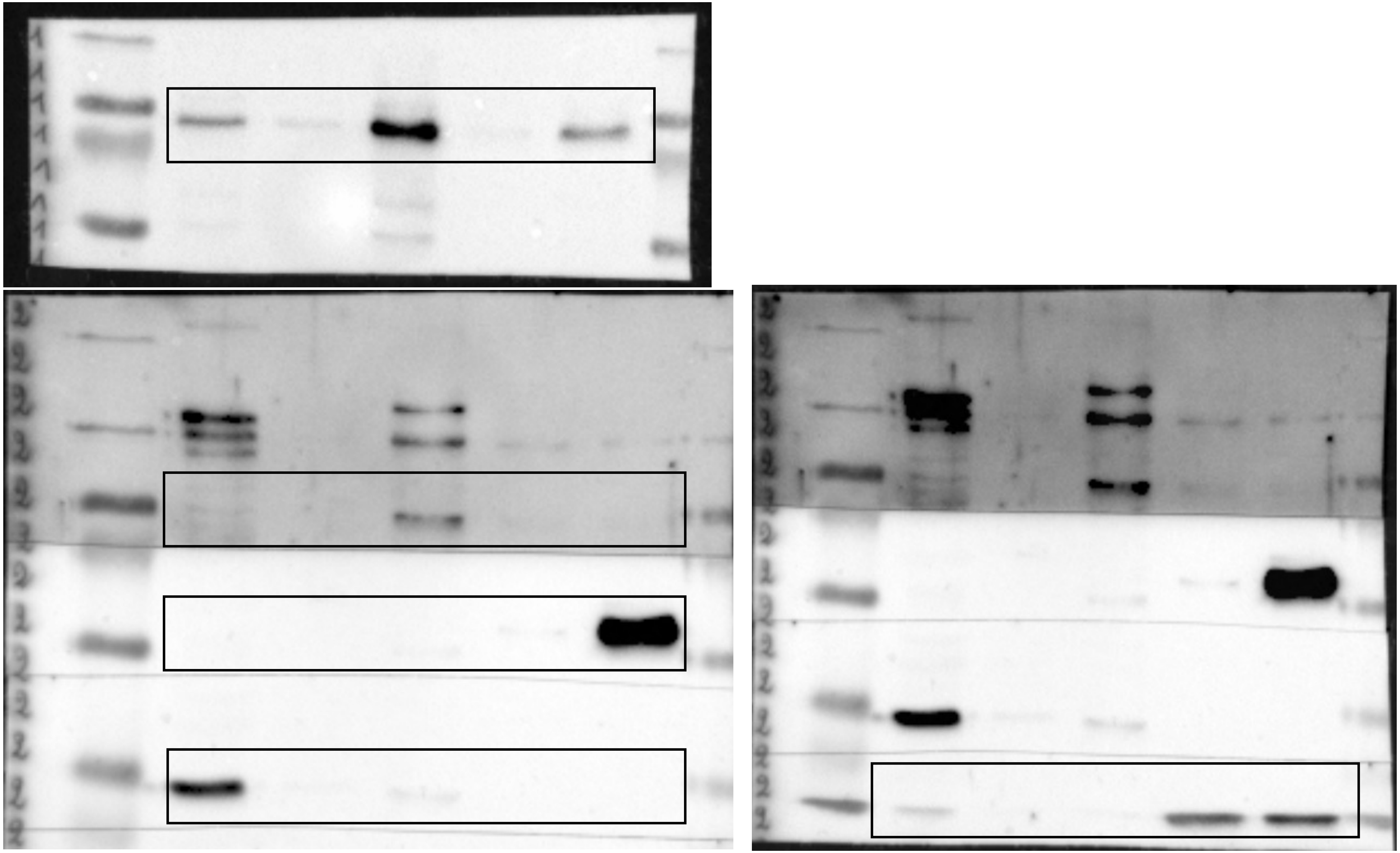

Supplementary Figure S3B

SLFN5

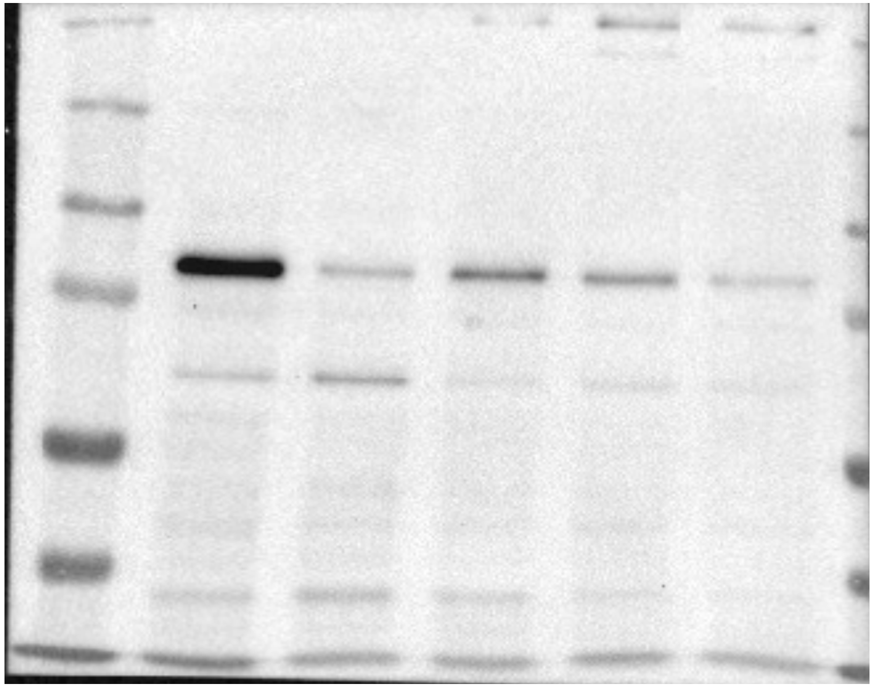

actin

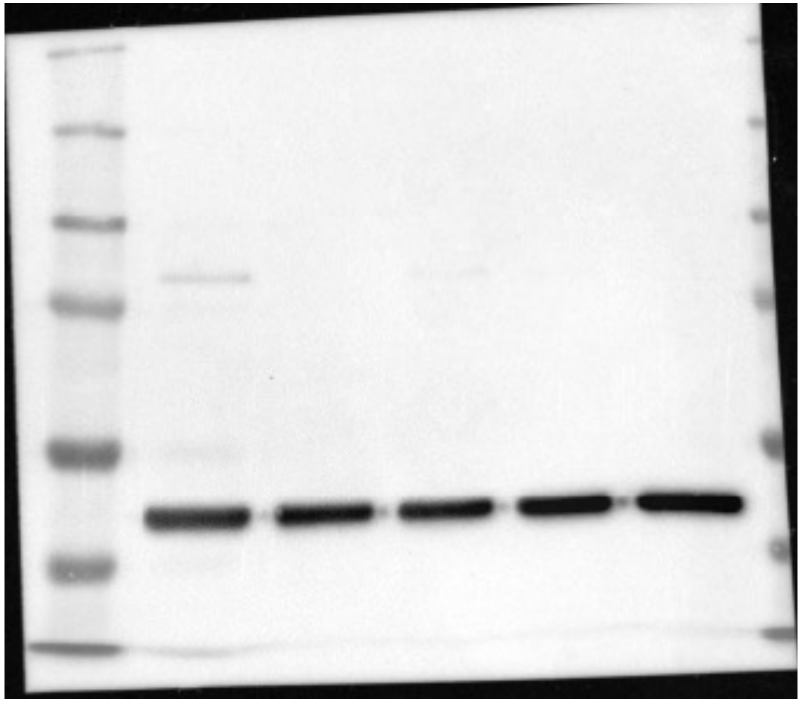

Supplementary Figure S3D

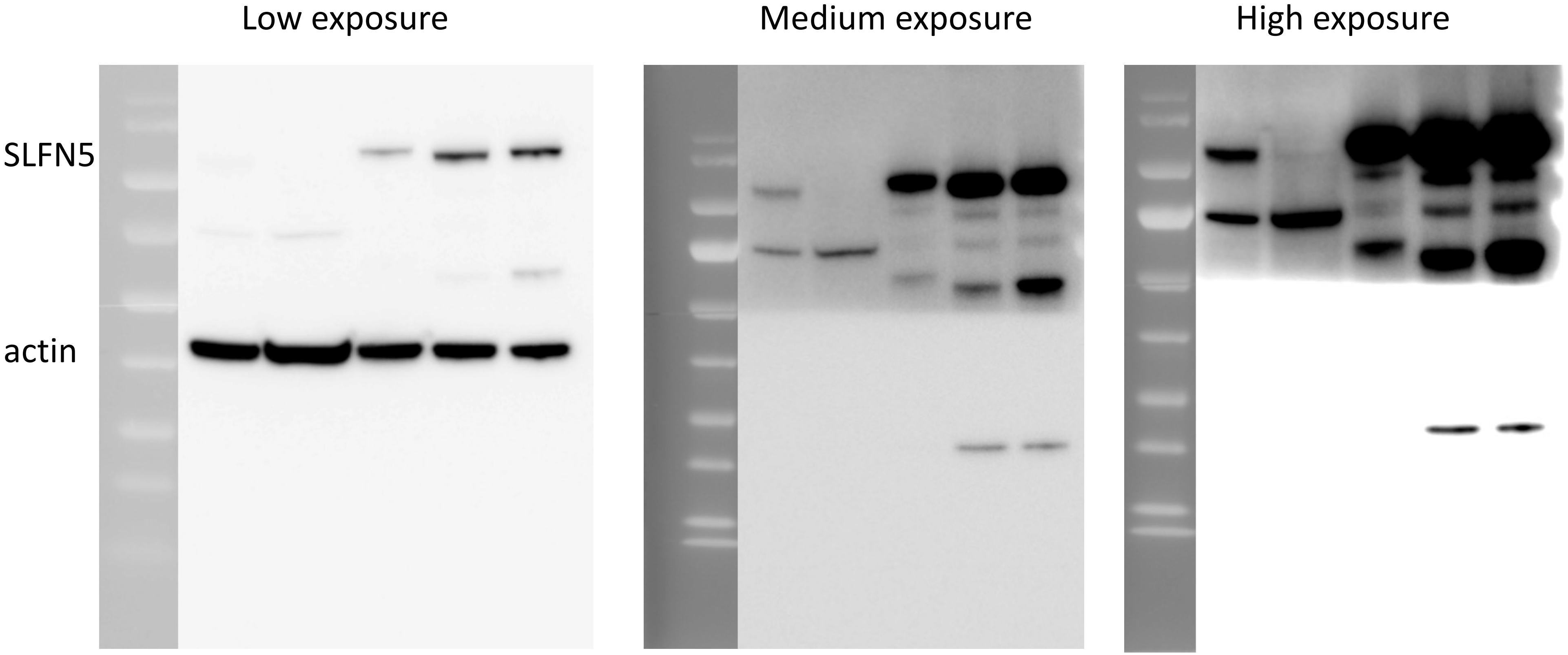

Supplementary Figure S4

A

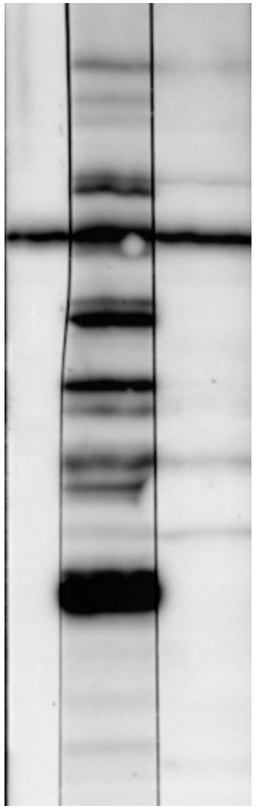

B

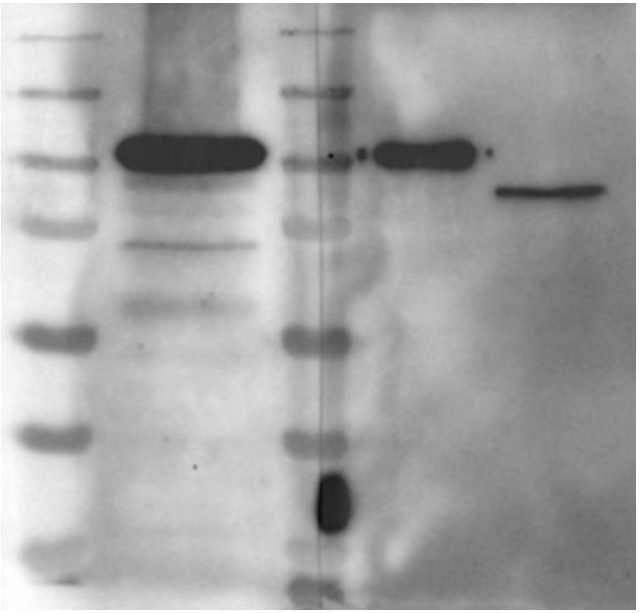

C

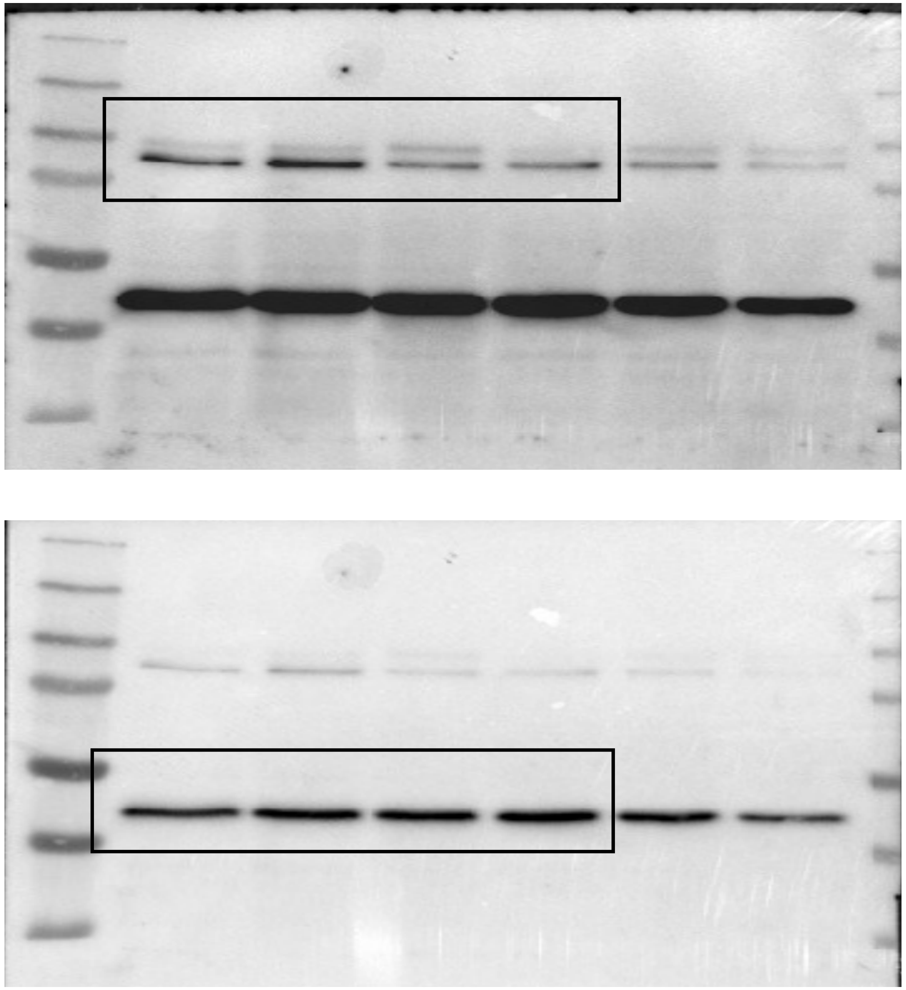

Supplementary Figure S4

D

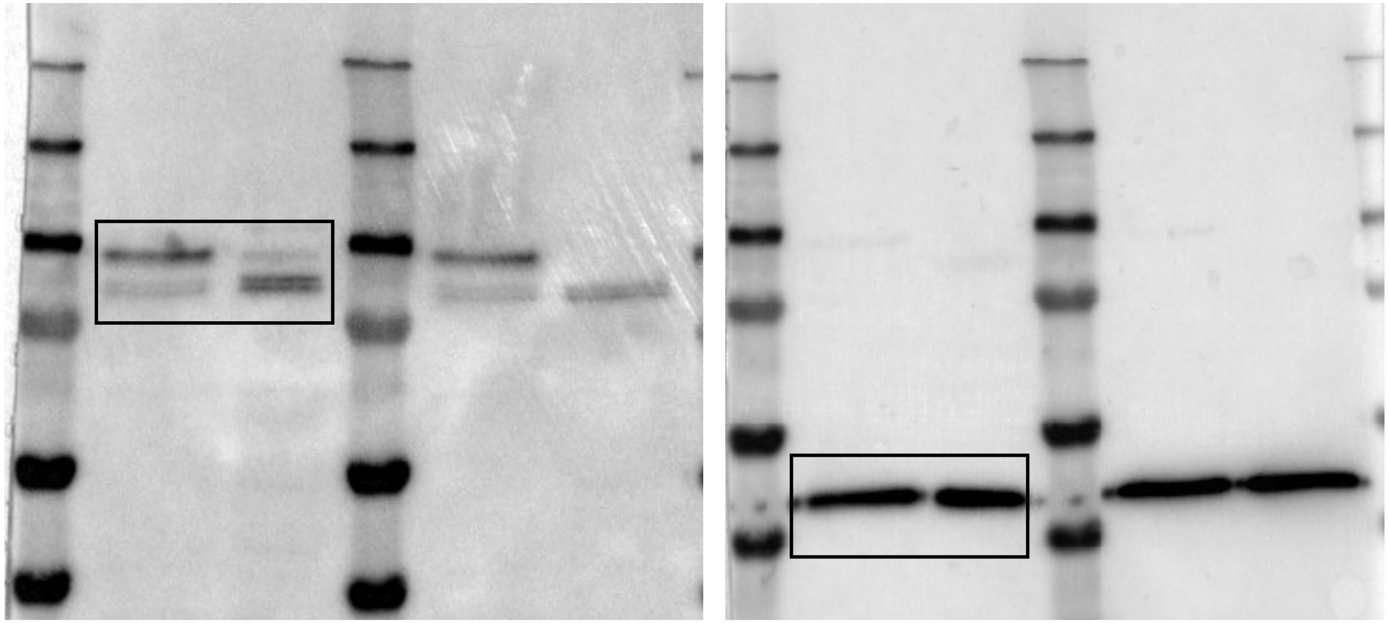

F

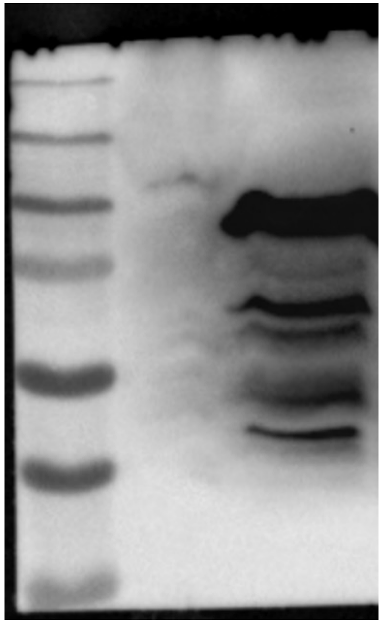

Supplementary Figure S2A

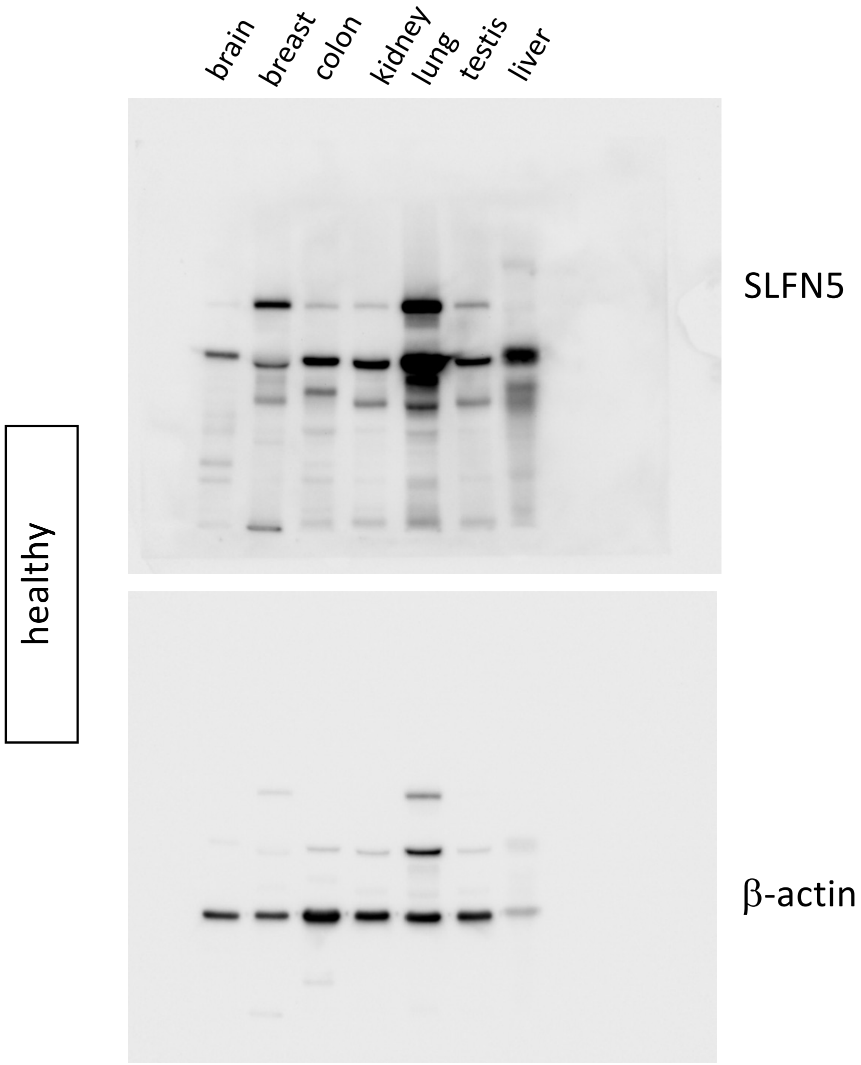

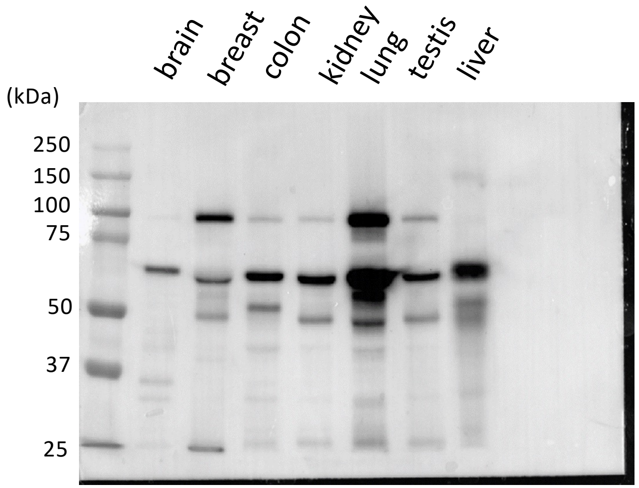

Supplementary Figure S2A

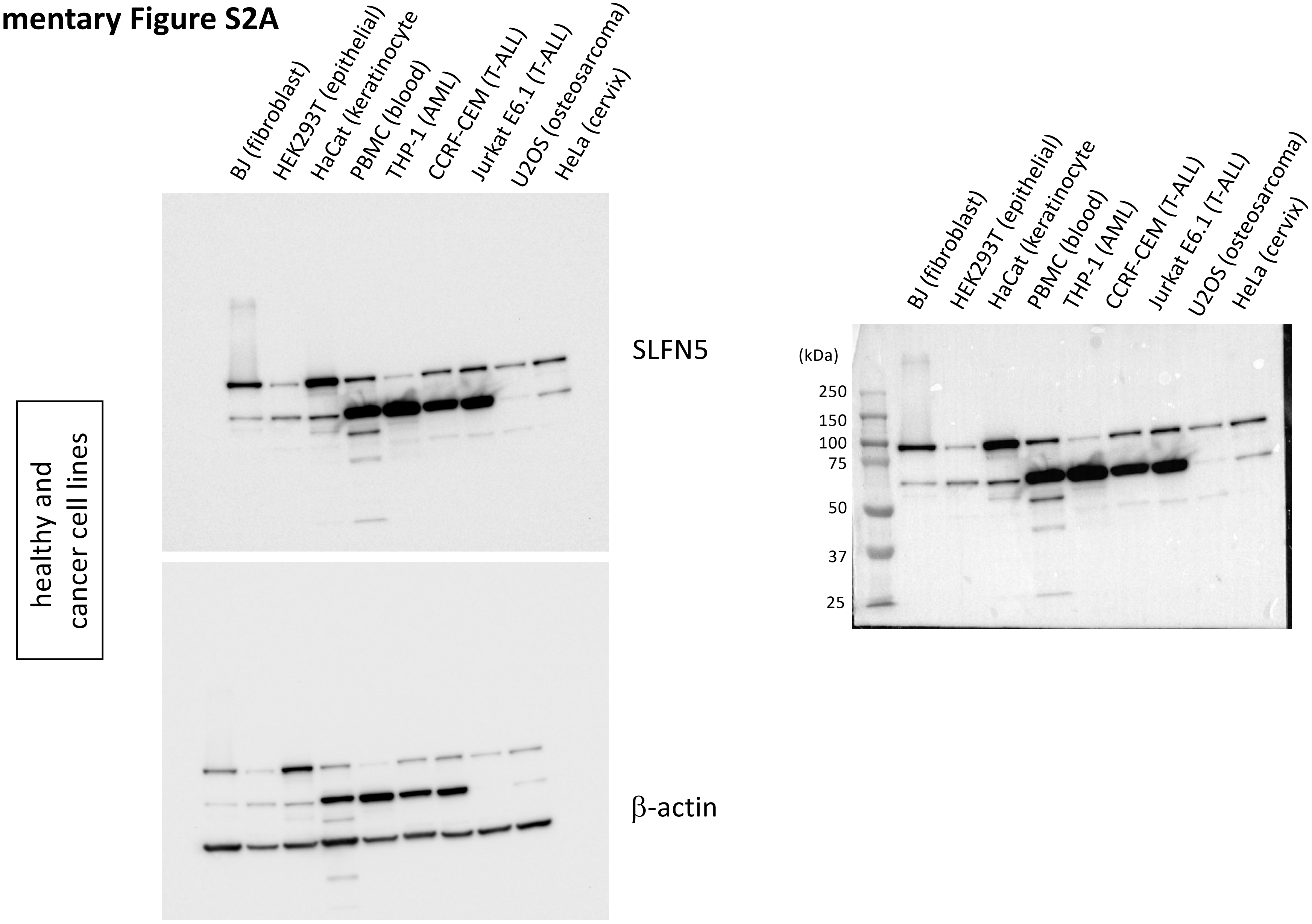

Supplementary Figure S2A

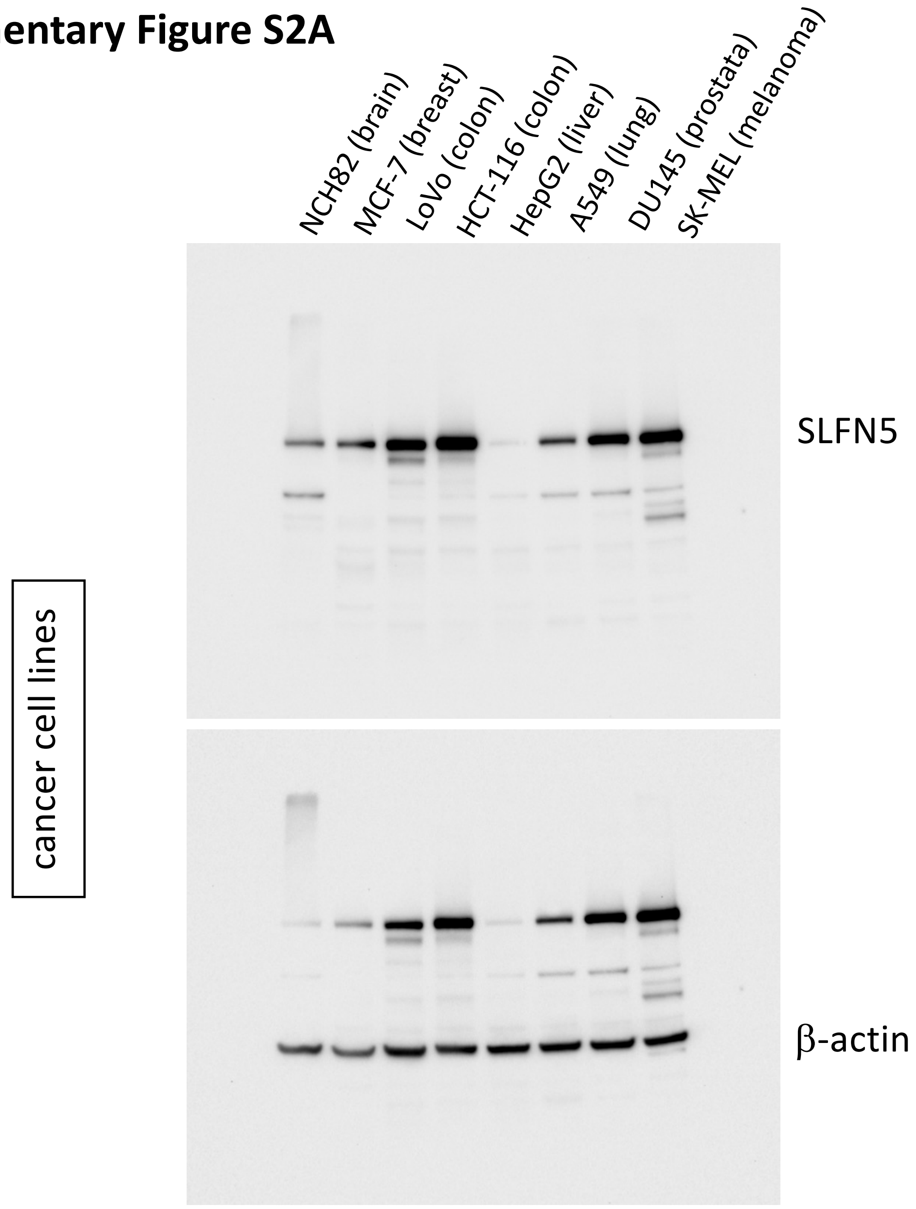

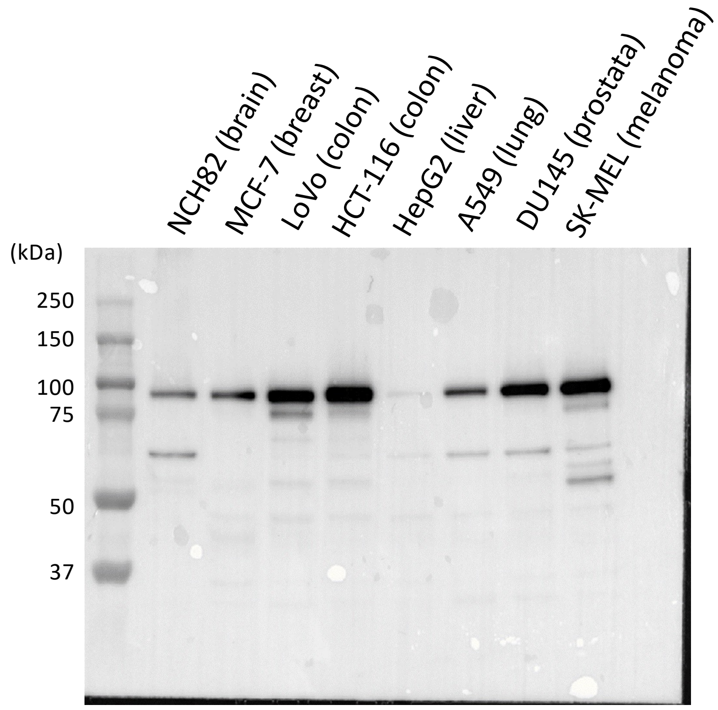

Supplementary Figure S2B

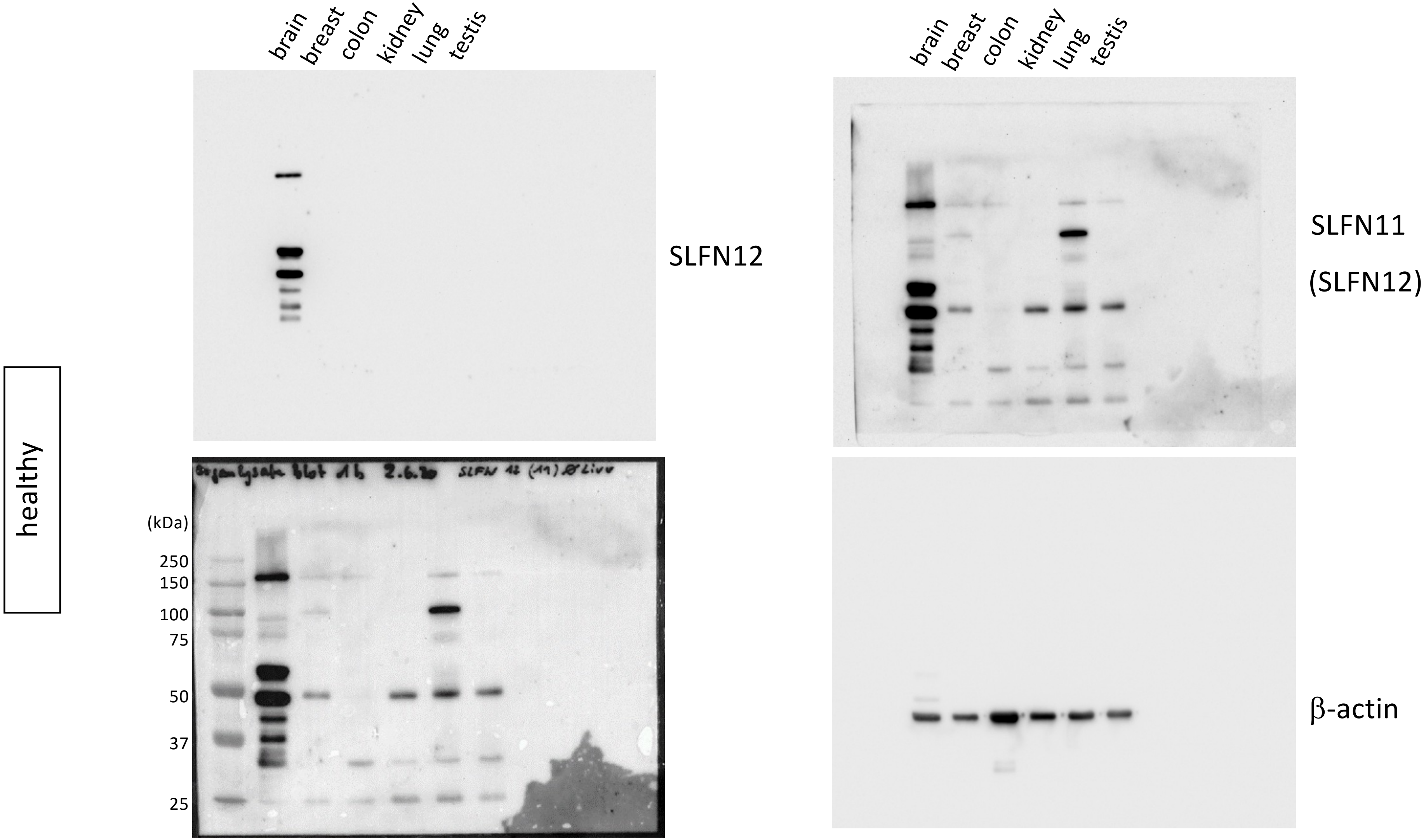

Supplementary Figure S2B

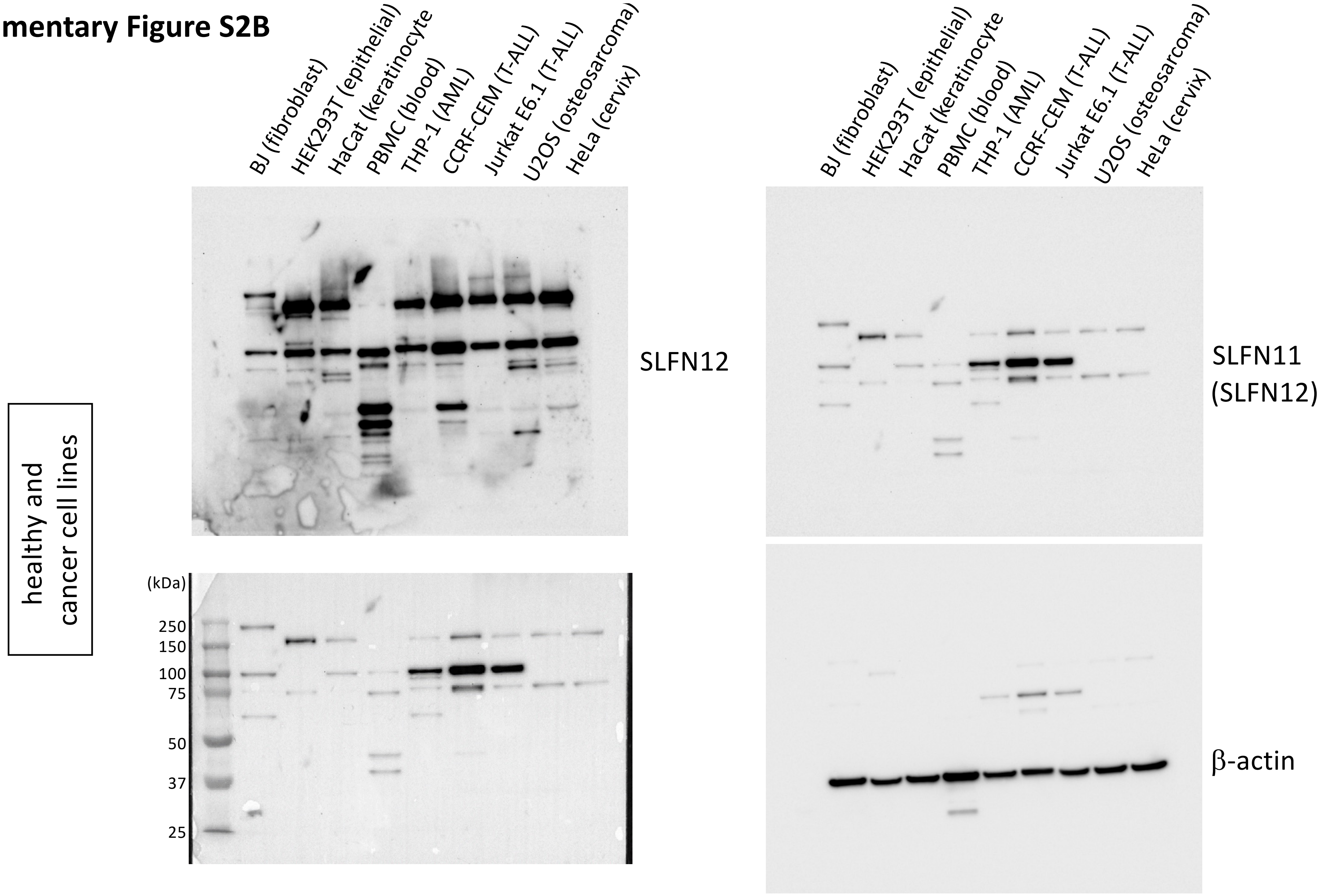

Supplementary Figure S2B

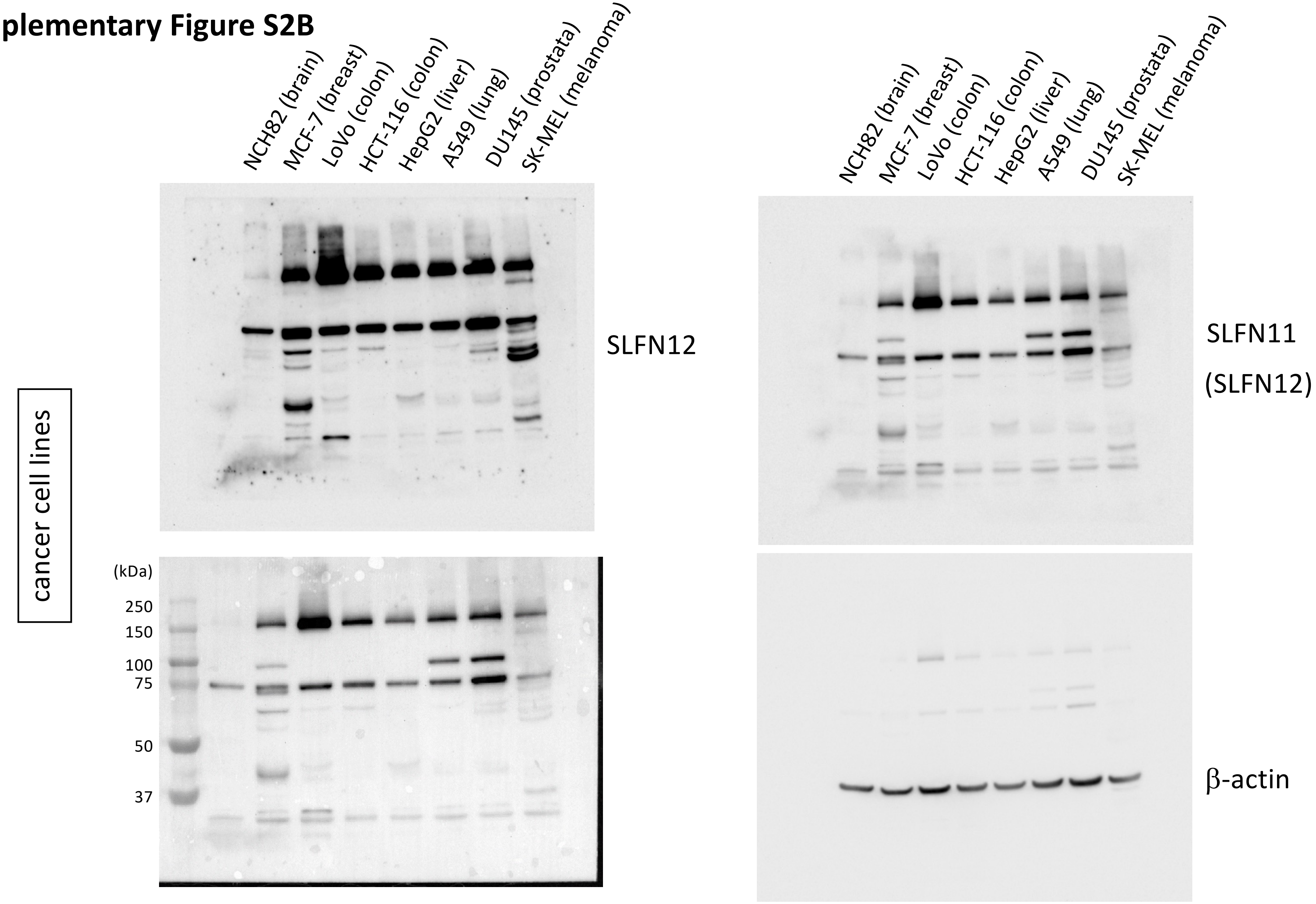

Supplement: Supplementary file 7 — Original Data File [file 41420_2022_1274_MOESM7_ESM.pdf]
